# Upregulated YB-1 protein promotes glioblastoma growth through a YB-1/CCT4/mLST8/mTOR pathway

**DOI:** 10.1172/JCI146536

**Published:** 2022-04-15

**Authors:** Jin-Zhu Wang, Hong Zhu, Pu You, Hui Liu, Wei-Kang Wang, Xiaojuan Fan, Yun Yang, Keren Xu, Yingfeng Zhu, Qunyi Li, Ping Wu, Chao Peng, Catherine C.L. Wong, Kaicheng Li, Yufeng Shi, Nu Zhang, Xiuxing Wang, Rong Zeng, Ying Huang, Liusong Yang, Zefeng Wang, Jingyi Hui

**Affiliations:** 1 State Key Laboratory of Molecular Biology, Shanghai Institute of Biochemistry and Cell Biology, CAS Center for Excellence in Molecular Cell Science, Chinese Academy of Sciences, University of Chinese Academy of Sciences, Shanghai, China.; 2 Shanghai Research Center for Brain Science and Brain-Inspired Intelligence, Institute of Brain-Intelligence Technology, Zhangjiang Laboratory, Shanghai, China.; 3 CAS Key Laboratory of Computational Biology, Shanghai Institute of Nutrition and Health and; 4 CAS Key Laboratory of Systems Biology, Shanghai Institute of Biochemistry and Cell Biology, CAS Center for Excellence in Molecular Cell Sciences, Chinese Academy of Sciences, University of Chinese Academy of Sciences, Shanghai, China.; 5 Department of Pathology and; 6 Department of Pharmacy, Huashan Hospital, Shanghai Medical College, Fudan University, Shanghai, China.; 7 National Facility for Protein Science in Shanghai, Zhangjiang Lab, Shanghai Advanced Research Institute, Chinese Academy of Science, Shanghai, China.; 8 Tongji University Cancer Center, Shanghai Tenth People’s Hospital of Tongji University, Center for Brain and Spinal Cord Research, School of Medicine, School of Life Sciences and Technology, Tongji University, Shanghai, China.; 9 Department of Neurosurgery, Institute of Precision Medicine, The First Affiliated Hospital of Sun Yat-sen University, Guangdong Provincial Key Laboratory of Brain Function and Disease, Guangzhou, Guangdong, China.; 10 School of Basic Medical Science, Nanjing Medical University, Nanjing, China.; 11 Department of General Surgery, Shanghai Key Laboratory of Biliary Tract Disease Research, State Key Laboratory of Oncogenes and Related Genes, Xinhua Hospital, Shanghai Jiao Tong University, Shanghai, China.; 12 Department of Neurosurgery, Huashan Hospital, Shanghai Medical College, Fudan University, Shanghai, China.

**Keywords:** Oncology, Molecular biology, Signal transduction, Translation

## Abstract

Y-box–binding protein 1 (YB-1) is a multifunctional RNA binding protein involved in virtually every step of RNA metabolism. However, the functions and mechanisms of YB-1 in one of the most aggressive cancers, glioblastoma, are not well understood. In this study, we found that YB-1 protein was markedly overexpressed in glioblastoma and acted as a critical activator of both mTORC1 and mTORC2 signaling. Mechanistically, YB-1 bound the 5′UTR of *CCT4* mRNA to promote the translation of CCT4, a component of the CCT chaperone complex, that in turn activated the mTOR signaling pathway by promoting mLST8 folding. In addition, YB-1 autoregulated its own translation by binding to its 5′UTR, leading to sustained activation of mTOR signaling. In patients with glioblastoma, high protein expression of YB-1 correlated with increased expression of CCT4 and mLST8 and activated mTOR signaling. Importantly, the administration of RNA decoys specifically targeting YB-1 in a mouse xenograft model resulted in slower tumor growth and better survival. Taken together, these findings uncover a disrupted proteostasis pathway involving a YB-1/CCT4/mLST8/mTOR axis in promoting glioblastoma growth, suggesting that YB-1 is a potential therapeutic target for the treatment of glioblastoma.

## Graphical abstract

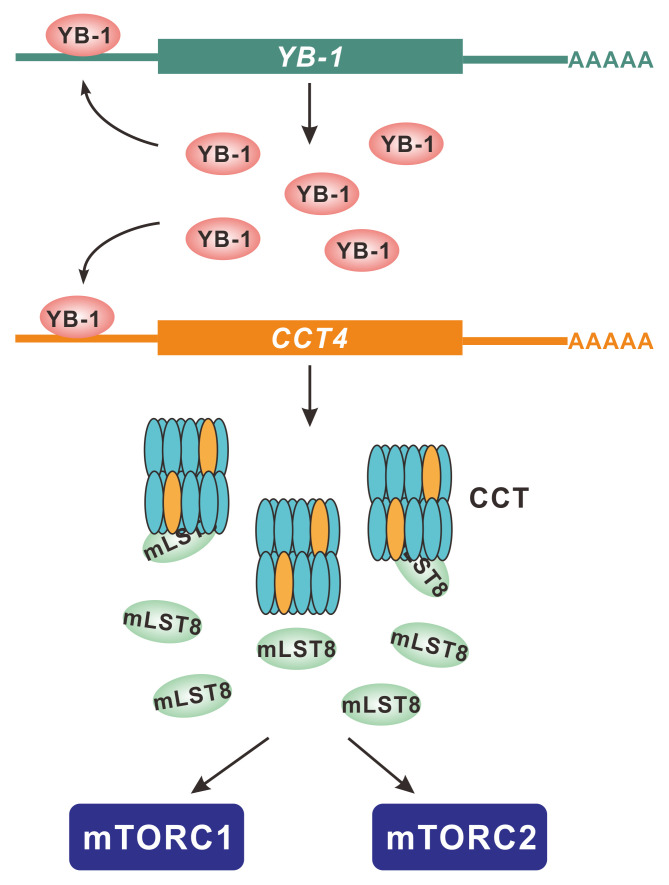

## Introduction

Recent large-scale surveys of proteins identified more than 1500 RNA binding proteins that bind to single- or double-stranded RNAs (1–4). RNA binding proteins together with different classes of RNAs form dynamic ribonucleoprotein (RNP) complexes in cells to determine the fates and functions of various RNAs (5). Dysregulation of RNA binding proteins has been linked to various human diseases, especially in cancers where a number of RNA binding proteins function as master regulators during cancer development and progression (6, 7). However, our current understanding of the molecular roles of RNA binding proteins in cancer remains very limited.

Gliomas are the most prevalent primary brain tumors in adults, and glioblastoma accounts for approximately 60% to 70% of malignant gliomas (8, 9). The majority of patients with glioblastoma receive surgical resection followed by radiation and chemotherapy (10, 11). Despite these therapeutic interventions, glioblastoma is associated with a poor prognosis, with a 5-year survival rate of 5.1% and a median survival of 15 to 23 months (12, 13). Some new investigational treatments, including targeted, radio-, chemo-, and immunotherapies, only yielded limited improvement of patient outcome (14). Therefore, a better understanding of the key molecules and pathways that trigger glioblastoma is warranted, which will facilitate the identification of novel targets for early diagnosis and effective treatment of glioblastoma.

The cold shock domain (CSD) is an evolutionarily conserved nucleic acid binding domain, which carries 2 consensus motifs found in a typical RNA recognition motif (RRM), RNP1 and RNP2 (15). The human Y-box–binding protein 1 (YB-1) is a key member of the mammalian CSD-containing protein family, which has been implicated in a wide variety of cellular processes under physiological and pathological conditions (16, 17). Through recognizing RNA, YB-1 has been suggested to participate in virtually all RNA-associated processes, including RNA splicing (18–21), stability (22, 23), packaging (24), and translation (25–28), as well as sorting, displacing, and processing noncoding RNAs (29–31). The expression of YB-1 is developmentally regulated, with distinct patterns in various tissues (32). In brain, YB-1 is expressed in fetal brain tissues and lost during glial differentiation, while it is reexpressed in glioblastoma tissues (33). Recent studies indicate that high YB-1 expression in glioma is associated with increased cell proliferation, survival, migration, and resistance to temozolomide (29, 34–36), and suggest YB-1 as a potential biomarker for glioma progression (37). However, the molecular functions and regulatory mechanisms of YB-1 in glioblastoma are not well understood.

The mammalian target of rapamycin (mTOR), a member of the serine/threonine protein kinase family, plays a critical role in cell growth, survival, motility, and metabolism via the regulation of protein synthesis (38). mTOR participates in the formation of 2 functionally distinct complexes in mammals, mTORC1 and mTORC2 (39). In this study, we applied transcriptomic and proteomic approaches to search for the downstream targets and pathways of YB-1 in glioblastoma. We identified YB-1 as a critical activator of both mTORC1 and mTORC2 signaling via stabilizing mLST8. Our data showed that YB-1 enhances the translation of CCT4, a component of the protein chaperone complex CCT (chaperonin containing TCP-1, also known as T-complex protein-1 ring complex [TRiC]), that in turn facilitates mLST8 folding via the CCT complex. Furthermore, RNA decoy oligonucleotides specifically bound to YB-1 and inhibited tumor growth in a mouse xenograft model. Our work highlights targeting YB-1 as a potential effective strategy for the treatment of glioblastoma.

## Results

### The level of YB-1 protein, but not its mRNA, is dramatically elevated in glioblastoma tissues.

**Figure 1:**
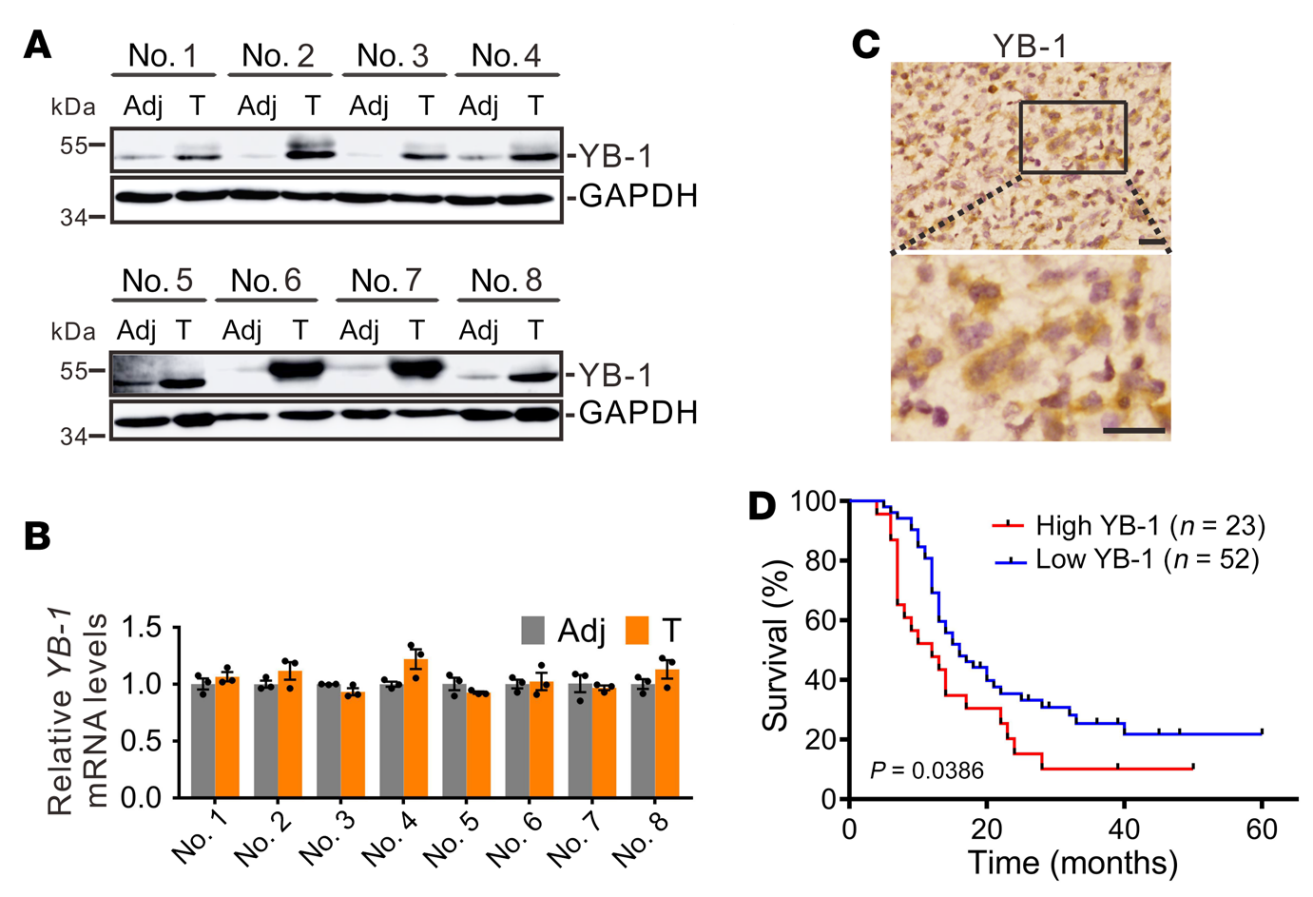
The protein level of YB-1 is substantially upregulated in glioblastoma, which predicts a poor prognosis. (**A**) Western blot analysis of YB-1 in 8 pairs of glioblastoma tissues (T) and their adjacent tissues (Adj). (**B**) RT-qPCR analysis of *YB-1* mRNA expression in 8 pairs of glioblastoma tissues and their adjacent tissues. Data are presented as mean ± SEM (*n =* 3). (**C**) Representative image of IHC staining of YB-1 in glioblastoma tissues. Scale bars: 20 μm. (**D**) Kaplan-Meier survival curves of glioblastoma patients with low (scores 0–150) versus high (scores 151–300) YB-1 expression. *P* value was determined by Mantel-Cox log-rank test.

To gain insights into the role of YB-1 in glioblastoma, we first examined the expression of YB-1 at both mRNA and protein levels in glioblastoma patient tissues. Consistent with previous results from mouse brain (33), YB-1 protein was expressed at a low level in normal glial tissues (Figure 1A). Surprisingly, the protein levels of YB-1 in 8 glioblastoma tissues were dramatically upregulated compared with those in paired adjacent tissues (Figure 1A), while the mRNA levels of *YB-1* did not show significant changes between glioblastoma and adjacent tissues (Figure 1B), suggesting that aberrant overexpression of YB-1 in glioblastoma predominantly takes place at the protein level. Immunohistochemical (IHC) staining of YB-1 was detected mainly in the cytoplasm of glioblastoma tissue cells (Figure 1C). Moreover, in a cohort of 75 patients with primary glioblastoma (Supplemental Table 1; supplemental material available online with this article; https://doi.org/10.1172/JCI146536DS1), higher protein expression of YB-1 predicts a poor prognosis (Figure 1D), supporting an oncogenic role of YB-1 in glioblastoma.

### Reduction of YB-1 in glioblastoma cells inhibits mTOR signaling.

**Figure 2:**
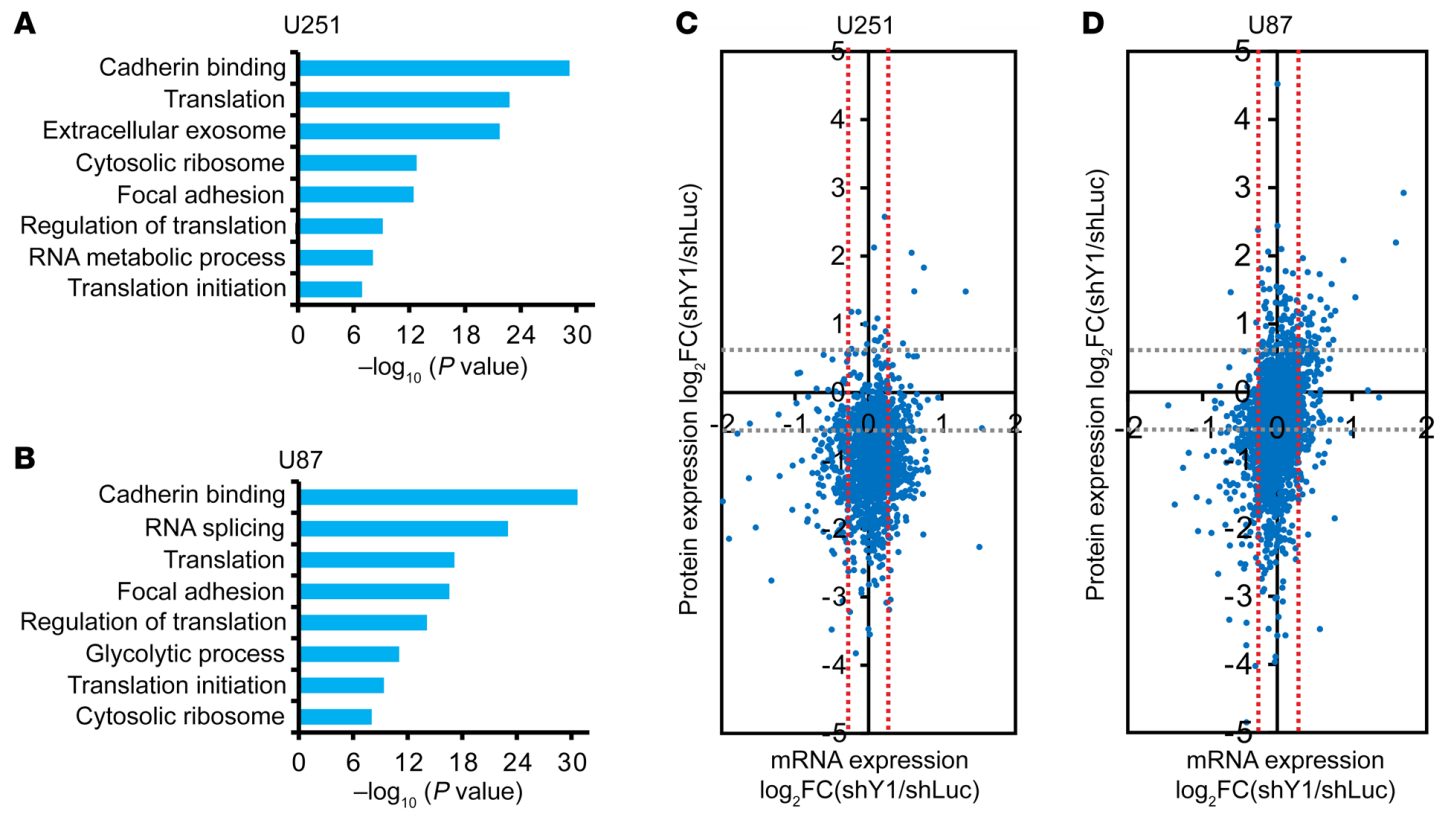
Transcriptomic and proteomic analyses of gene expression changes upon YB-1 deletion in glioblastoma cells. (**A** and **B**) GO enrichment analyses of significantly downregulated proteins in YB-1–depleted U251 (**A**) and U87 (**B**) cells. (**C** and **D**) Scatterplot integrating proteomic (*y* axis) and RNA-seq (*x* axis) data sets from U251 (**C**) or U87 (**D**) cells. Red dotted lines represent an absolute FC of 1.2 or –1.2 at the mRNA level, and gray dotted lines indicate an absolute FC of 1.5 or –1.5 at the protein level.

**Figure 3:**
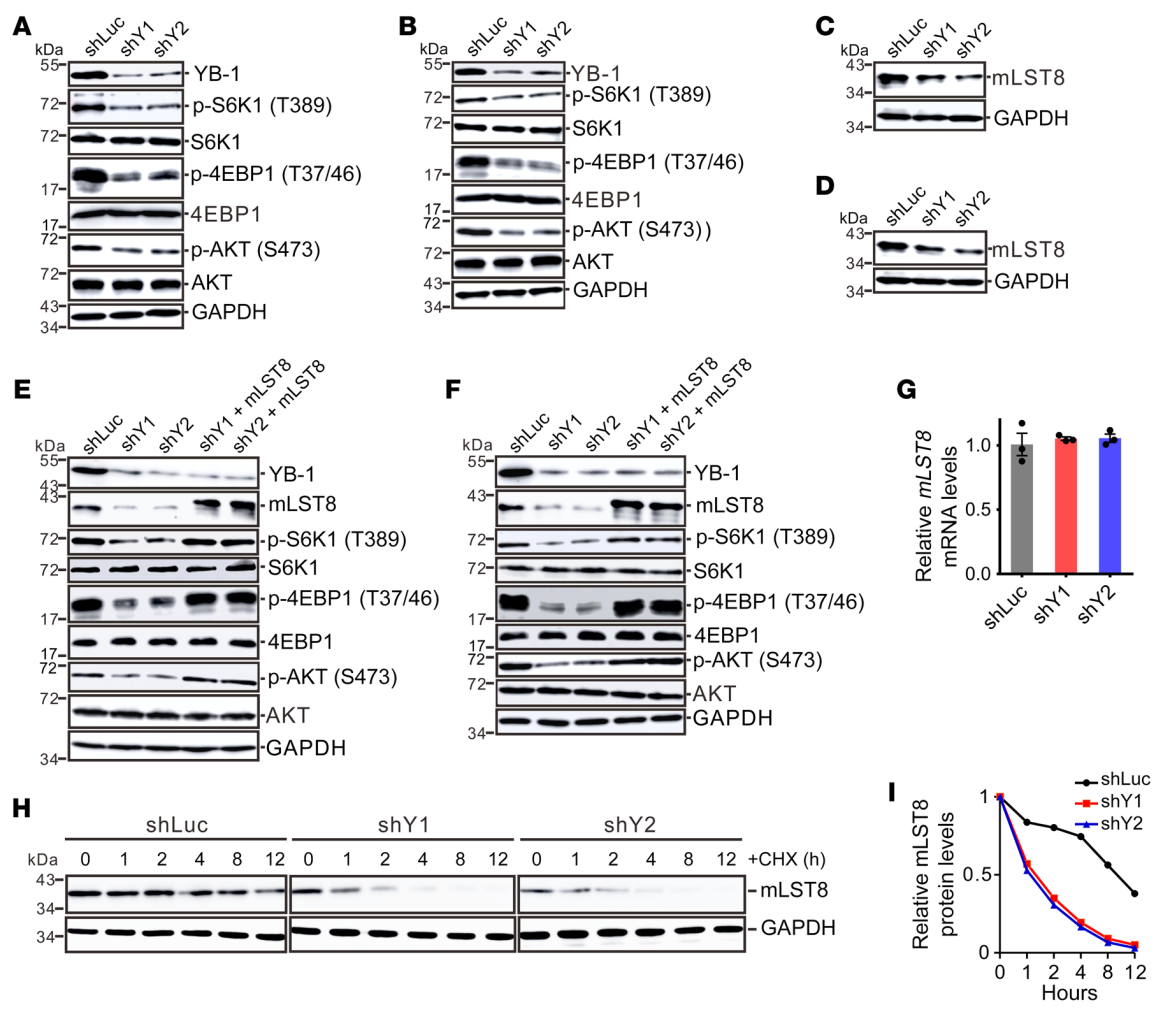
YB-1 activates both mTORC1 and mTORC2 signaling by stabilizing mLST8. (**A** and **B**) Western blot analysis of the molecular markers for mTORC1 and mTORC2 signaling in control or YB-1–knockdown U251 (**A**) and U87 (**B**) cells. (**C** and **D**) Western blot analysis of mLST8 in control or YB-1–knockdown U251 (**C**) and U87 (**D**) cells. (**E** and **F**) Western blot analysis of YB-1, mLST8, and mTOR markers in control, YB-1–knockdown cells, or YB-1 knockdown complemented with mLST8 in U251 (**E**) and U87 (**F**) cells. (**G**) RT-qPCR analysis of *mLST8* mRNA expression in control or YB-1–knockdown U251 cells. Data are presented as mean ± SEM (*n =* 3). (**H**) Western blot analysis of mLST8 in control or YB-1–knockdown U251 cells treated with CHX for the times indicated. Data represent 3 independent experiments. (**I**) Quantification of the relative mLST8 protein levels in **H**.

To investigate the functions and mechanisms of YB-1 in glioblastoma cells, we first established YB-1 knockdown in glioblastoma cell lines U251 and U87 by stably expressing 2 shRNAs targeting YB-1. Both shRNAs resulted in an efficient depletion of YB-1 in U251 and U87 cells (Supplemental Figure 1, A and B), and led to decreased cell proliferation, migration, and invasion (Supplemental Figure 1, C–H). These results suggested that overexpression of YB-1 may contribute to the highly proliferative, migratory, and invasive properties of glioblastoma. Next, we used multiomics approaches to identify YB-1–regulated pathways in glioblastoma. Transcriptomic analyses by RNA sequencing (RNA-seq) detected 227 and 208 upregulated genes and 550 and 199 downregulated genes (fold change [FC] > 2, FDR < 0.05) after YB-1 knockdown in U251 and U87 cells, respectively. Gene Ontology (GO) analyses (40, 41) showed that YB-1–regulated genes are enriched in the categories of nervous system development, neurogenesis, neuron differentiation, receptor ligand activity, and extracellular matrix organization (Supplemental Figure 2, A and B). Interestingly, the quantitative proteomic analysis showed that a much larger number of proteins are significantly downregulated in YB-1–knockdown cells compared with the number of upregulated proteins (FC > 1.5), i.e., 213 downregulated versus 2 upregulated in U251 cells and 467 downregulated versus 45 upregulated in U87 cells. The downregulated proteins are enriched in the GO categories of cadherin binding, translation, ribosome assembly, RNA processing, and focal adhesion in both cell lines (Figure 2, A and B). Furthermore, combining transcriptomic and proteomic data, we found that, among genes with little change at the mRNA level (FC < 1.2), after YB-1 depletion the majority of genes had decreased expression at the protein level in both cell lines (Figure 2, C and D), suggesting that YB-1 promotes the expression of a large number of genes at the translational level. We noticed that YB-1–upregulated proteins include a set of translation and ribosomal factors and components of glycolysis, autophagy, lipogenesis, and pentose phosphate pathways, which are known targets of mTOR signaling (42). We validated the mass spectrometry (MS) data by performing immunoblotting assays and found that YB-1 depletion repressed the expression of TPI1, PGAM1, PKM, G6PD, and FASN in both U251 and U87 cells (Supplemental Figure 2, C and D). Knockdown of YB-1 also reduced the p62 protein level, which is a signature for the activation of autophagy. Accordingly, YB-1 depletion resulted in the increase of LC3-II, another marker for autophagic activity (Supplemental Figure 2, C and D). Based on these results, we hypothesized that knockdown of YB-1 inhibits mTOR signaling. To test this idea, we examined several markers for mTORC1 (phospho-S6K1 T389 and phospho-4EBP1 T37/46) and mTORC2 (phospho-AKT S473). All these markers showed significant decrease in YB-1–knockdown U251 and U87 cells compared with control cells, indicating that both mTORC1 and mTORC2 signaling was repressed when YB-1 expression was inhibited (Figure 3, A and B).

### YB-1 activates both mTORC1 and mTORC2 signaling through mLST8.

Since YB-1 is capable of upregulating both mTORC1 and mTORC2 signaling, we first speculated that YB-1 stimulates the expression of a common component shared by both mTOR complexes. Besides mTOR protein, mTORC1 contains mammalian lethal with SEC13 protein 8 (mLST8), DEP domain–containing mTOR-interacting protein (DEPTOR), and regulatory associated protein of mTOR (RAPTOR), while mTORC2 consists of mLST8, DEPTOR, rapamycin-insensitive companion of mTOR (RICTOR), and mammalian stress-activated MAP kinase–interacting protein 1 (mSIN1). We tested these proteins by immunoblotting and found that the shared component of both mTOR complexes, mLST8, was repressed after knockdown of YB-1 in U251 and U87 cells (Figure 3, C and D), while the expression of other major mTOR components did not show apparent changes (Supplemental Figure 2, E and F). mLST8 was identified as a member of mTOR pathway that binds and stimulates mTOR kinase activity (43). Consistently, ectopic expression of mLST8 in YB-1–knockdown cells rescued the attenuated signaling activity of both mTORC1 and mTORC2 (Figure 3, E and F).

We further examined how YB-1 controls the expression of mLST8. Real-time quantitative reverse transcription polymerase chain reaction (RT-qPCR) analysis showed that the mRNA level of *mLST8* was not affected by YB-1 (Figure 3G), suggesting that YB-1 posttranscriptionally controls mLST8 expression. To test whether YB-1 regulates the translation of *mLST8* mRNA, we performed a polysome profiling assay and did not observe apparent changes in *mLST8* mRNA abundance upon YB-1 knockdown in sucrose gradient fractions corresponding to different stages during active translation (Supplemental Figure 3, A–C). We therefore hypothesized that YB-1 might play a role in stabilizing the mLST8 protein. To test this hypothesis, we investigated the stability of mLST8 in the control or YB-1–knockdown cells treated with the translation inhibitor cycloheximide (CHX) and found that knockdown of YB-1 led to faster degradation of mLST8 (Figure 3, H and I). We further examined which protein degradation pathway is involved in mLST8 destabilization after YB-1 knockdown using different inhibitors. Our results showed that the lysosome inhibitor bafilomycin A1 (Baf A1), but not the proteasome inhibitor MG132, rescued the protein level of mLST8 in YB-1–knockdown cells (Supplemental Figure 4, A and B), suggesting that YB-1 depletion induced lysosome-mediated protein degradation of mLST8. Together, these data indicate that YB-1 upregulates both mTORC1 and mTORC2 signaling by stabilizing mLST8 protein.

### YB-1 stabilizes mLST8 protein via increasing CCT4 mRNA translation.

**Figure 4:**
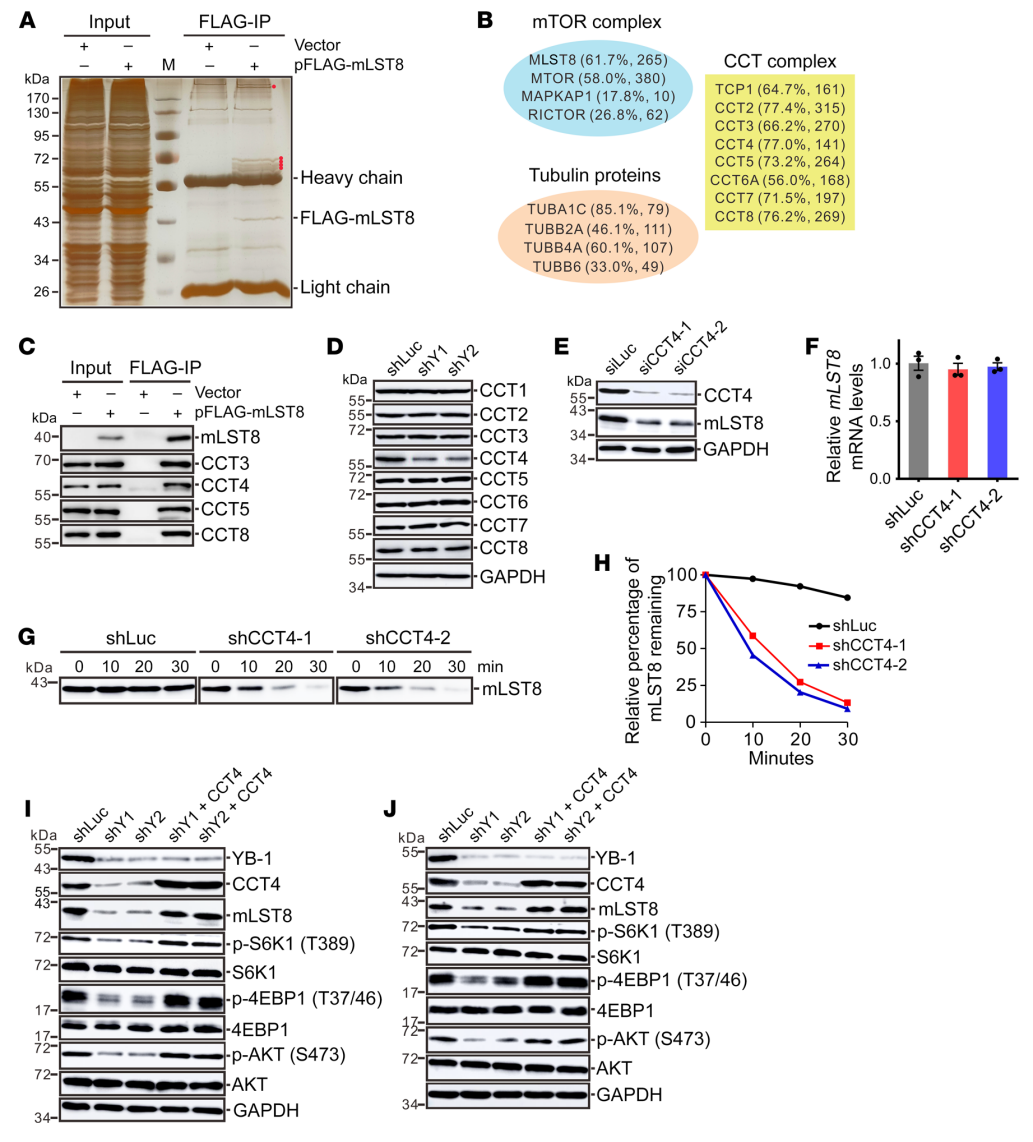
YB-1 promotes mLST8 folding via CCT4. (**A**) Immunopurification of mLST8-interacting proteins from U251 cells stably expressing FLAG-tagged mLST8 followed by SDS-PAGE and visualization with silver staining. The major specific interacting proteins are indicated by red dots. (**B**) The components of mTOR and CCT complexes and tubulin proteins were identified by mass spectrometry as mLST8-interacting proteins with high confidence. The percentage of peptide coverage and the number of peptide spectra matched for the protein are shown in the parentheses. (**C**) Immunoprecipitation of CCT components from HEK293T cells transiently transfected with a vector or FLAG-tagged mLST8 expression construct using an anti-FLAG antibody followed by immunoblotting analysis. (**D**) Western blot analysis of CCT components in control or YB-1–knockdown U251 cells. (**E**) Western blot analysis of CCT4 and mLST8 in U251 cells transfected with control or CCT4-specific siRNA. (**F**) RT-qPCR analysis of *mLST8* mRNA in U251 cells expressing control or CCT4-specific shRNA. Data are presented as mean ± SEM (*n =* 3). (**G**) Western blot analysis of mLST8 in control or CCT4-knockdown U251 cells treated with Baf A1 followed by incubation with thermolysin for the indicated time. Data represent 3 independent experiments. (**H**) Quantitation of **G**. (**I** and **J**) Western blot analysis of YB-1, CCT4, mLST8, and the markers for mTORC1 and mTORC2 signaling in control, YB-1–knockdown, or YB-1 knockdown complemented with CCT4 in U251 (**I**) and U87 (**J**) cells.

To further examine how YB-1 safeguards mLST8 protein, we searched for mLST8 interacting proteins that may regulate the stability of mLST8. We performed immunoprecipitation using anti-FLAG antibody from cells stably expressing FLAG-tagged mLST8 followed by MS analysis (Figure 4A). Besides mTOR components mTOR, RICTOR, and mSIN1 (MAPKAP1), all components of a chaperone complex, CCT and tubulin proteins were recovered with high confidence (Figure 4B). Interestingly, tubulin proteins are well-studied substrates of the CCT complex (44). These data suggested that the CCT complex may participate in the regulation of mLST8 stability.

The CCT complex is composed of 8 proteins (CCT1–8, CCT1 is named TCP1 in humans), and plays critical roles in regulating cellular proteostasis (45). We first verified the interactions between mLST8 and CCT components by immunoprecipitation using FLAG-tagged mLST8 as a bait (Figure 4C), suggesting that mLST8 is a potential substrate of the CCT complex. Next, we hypothesized that YB-1 might affect the stability of mLST8 by regulating the expression of CCT proteins. Indeed, among the 8 components of the CCT complex, CCT4 was markedly repressed upon knockdown of YB-1, whereas subtle effects were observed for other CCT components (Figure 4D). Notably, knockdown of CCT4 suppressed mLST8 protein expression without changing the mRNA level of *mLST8* (Figure 4, E and F). Treatment of CCT4-knockdown cells with Baf A1 but not MG132 rescued mLST8 protein levels (Supplemental Figure 4, C and D), indicating that CCT4 protected mLST8 from lysosomal degradation. Because the CCT complex has been shown to function in protein folding, we hypothesized that downregulation of CCT4 might induce malfunction of the CCT complex, in turn leading to misfolding of mLST8 and its degradation by lysosomes. Thermolysin is a proteinase that was previously shown to preferentially degrade unfolded proteins (46). We found that the mLST8 protein protected by Baf A1 treatment was more sensitive to thermolysin in CCT4-knockdown cells compared with Baf A1–treated control cells (Figure 4, G and H). These results indicated that CCT4 promotes appropriate folding of mLST8 and protects it from degradation induced by misfolding. In addition, ectopically expressing CCT4 in YB-1–knockdown cells rescued repressed signaling activity of mTORC1 and mTORC2 (Figure 4, I and J). These data indicated that CCT4 facilitates efficient folding of mLST8 by the CCT complex and YB-1 upregulates mTOR signaling through the CCT4/mLST8 cascade.

### YB-1 promotes CCT4 mRNA translation by interacting with its 5′UTR.

**Figure 5:**
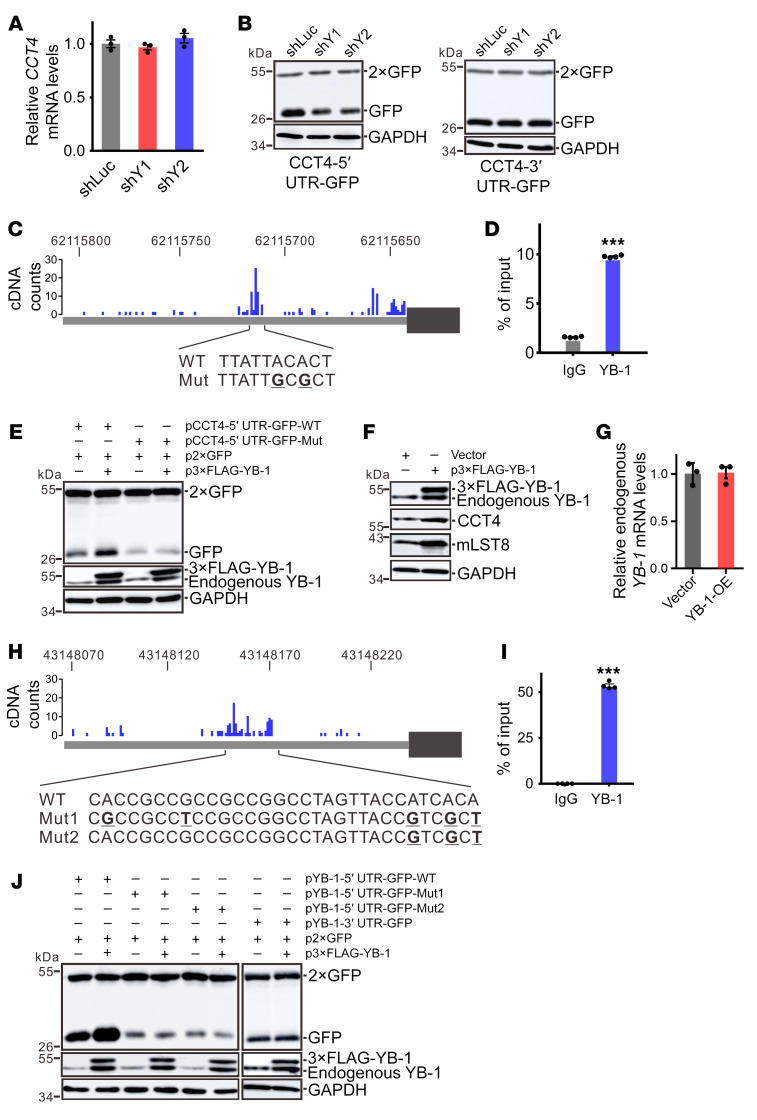
YB-1 regulates the translation of *CCT4* and its own mRNAs. (**A**) RT-qPCR analysis of *CCT4* mRNA in control or YB-1–knockdown U251 cells. Data are presented as mean ± SEM (*n =* 3). (**B**) Western blot analysis of GFP expression in control or YB-1–knockdown U251 cells transfected with *CCT4* 5′UTR (left) and 3′UTR (right) reporters and p2×GFP. p2×GFP serves as a transfection control. (**C**) iCLIP cDNA counts for YB-1 binding sites in the *CCT4* 5′UTR. The wild-type and substituted sequences in reporter constructs are shown below the schematic representation of the *CCT4* 5′UTR. (**D**) CLIP–RT-qPCR analysis of YB-1 binding to the *CCT4* 5′UTR. Data are presented as mean ± SEM (*n =* 4). ***P < 0.001 by unpaired, 2-tailed Student’s *t* test. (**E**) Western blot analysis of GFP expression in HEK293T cells transfected with indicated plasmids. p2×GFP serves as a transfection control. (**F**) Western blot analysis of YB-1, CCT4, and mLST8 in U251 cells expressing 3×FLAG-tagged YB-1 protein. (**G**) RT-qPCR analysis of endogenous *YB-1* mRNA in HEK293T cells transfected with empty vector or 3×FLAG-tagged YB-1 expression construct. Data are presented as mean ± SEM (*n =* 3). (**H**) iCLIP cDNA counts for YB-1 binding sites in the *YB-1* 5′UTR. The wild-type and substituted sequences in reporter constructs are shown below the schematic representation of the *YB-1* 5′UTR. (**I**) CLIP–RT-qPCR analysis of YB-1 binding to the *YB-1* 5′UTR. Data are presented as mean ± SEM (*n =* 4). ***P < 0.001 by unpaired, 2-tailed Student’s *t* test. (**J**) Western blot analysis of GFP expression in HEK293T cells transfected with indicated plasmids. p2×GFP serves as a transfection control.

To mechanistically understand how YB-1 upregulates CCT4 protein expression without changing the mRNA level of *CCT4* (Figure 5A), we further determined the association of *CCT4* mRNA with ribosomes and polysomes using sucrose gradient fractionation. We found a decrease in *CCT4* transcripts in polysome fractions of YB-1–knockdown cells compared with control cells, suggesting that CCT4 translation initiation was blocked by depletion of YB-1 (Supplemental Figure 3). We performed reporter assays using GFP expression constructs carrying the 5′UTR or 3′UTR sequence of *CCT4* cloned upstream or downstream of the GFP coding region. A construct expressing 2 copies of GFP protein (p2×GFP) served as a transfection control. YB-1 knockdown reduced the expression of the 5′UTR reporter, but not that of the 3′UTR reporter (Figure 5B). To examine whether YB-1 regulates *CCT4* translation by binding to its 5′UTR, we reanalyzed our previous individual nucleotide resolution cross-linking and immunoprecipitation combined with sequencing (iCLIP-seq) data from U251 cells that provided information on genome-wide mapping of in vivo YB-1 binding sites (29) and found a binding peak in the 5′UTR of *CCT4* (Figure 5C). CLIP–RT-qPCR analysis confirmed YB-1 binding to the *CCT4* 5′UTR (Figure 5D). Importantly, when we mutated this binding site, the *CCT4* 5′UTR mutant reporter was no longer sensitive to YB-1 overexpression (Figure 5E), indicating that YB-1 recognizes its binding site in the 5′UTR of *CCT4* mRNA to stimulate CCT4 translation.

### YB-1 autoregulates its own protein synthesis.

In Figure 5E, we made an intriguing observation that overexpression of YB-1 enhanced endogenous YB-1 expression in HEK293T cells. Similarly, overexpression of YB-1 in U251 cells also stimulated the expression of endogenous YB-1 together with CCT4 and mLST8 (Figure 5F). RT-qPCR results showed that the mRNA level of endogenous *YB-1* was not affected by ectopically expressed YB-1 (Figure 5G), and thus we reasoned that YB-1 may be capable of upregulating the translation of its own mRNA. Combined with iCLIP-seq data and CLIP–RT-qPCR validation, we identified several YB-1 binding sites in the 5′UTR of *YB-1* mRNA (Figure 5, H and I). Mutation of the YB-1 binding site at the 3′ end of the CLIP peak in its 5′UTR resulted in decreased expression of the reporter gene and loss of response to YB-1 overexpression, while the *YB-1* 3′UTR did not respond to YB-1 overexpression (Figure 5J). Taken together, these data indicated that YB-1 activates the translation of its own mRNA and *CCT4* mRNA through binding to their 5′UTRs, forming a positive feedback that activates the CCT complex.

### YB-1 maintains the self-renewal of glioblastoma stem–like cells via the CCT4/mLST8 cascade.

**Figure 6:**
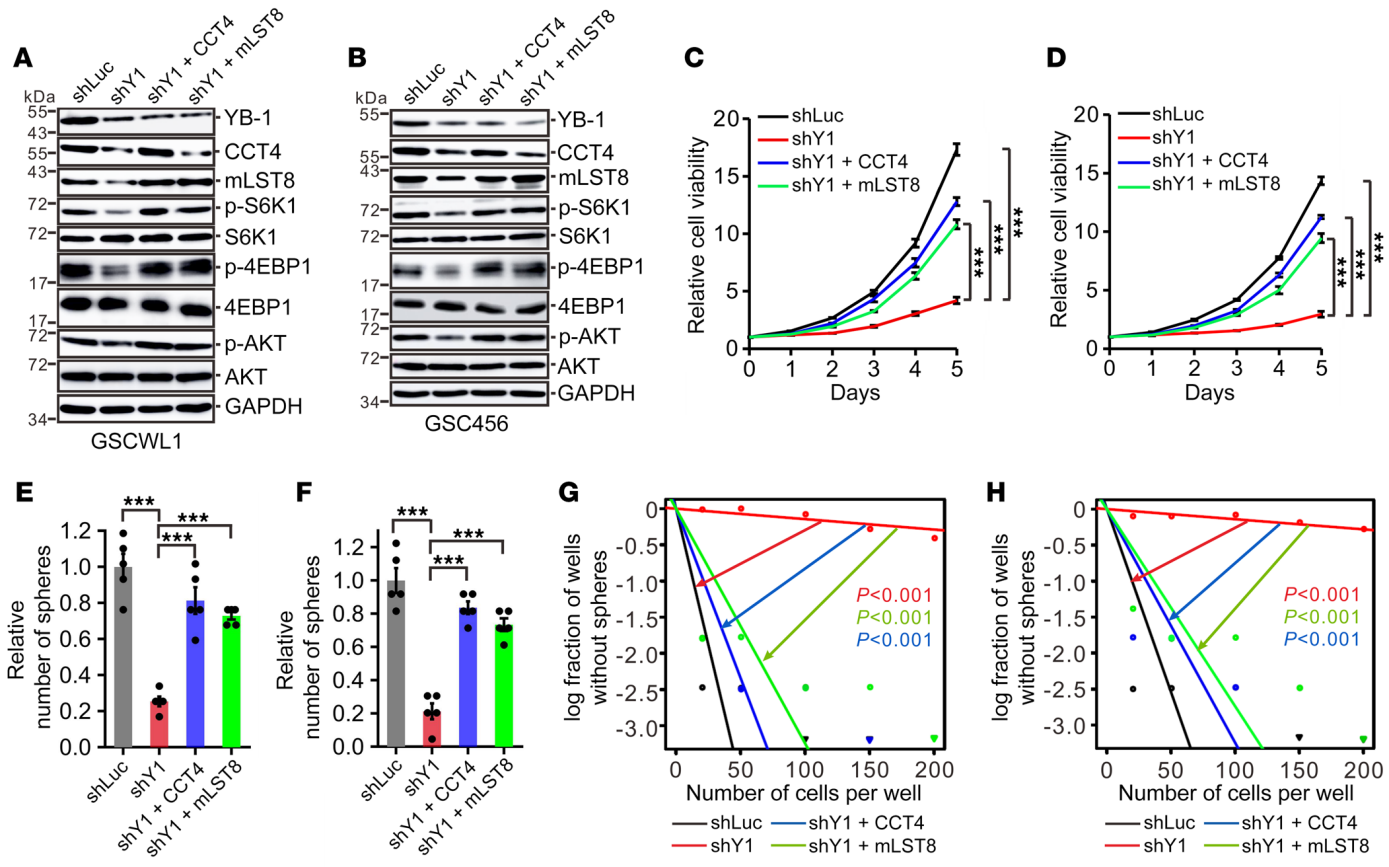
The YB-1/CCT4/mLST8 axis is required for cell proliferation and self-renewal of GSCs. (**A** and **B**) Western blot analysis of YB-1, CCT4, mLST8, and mTOR markers in GSCWL1 (**A**) and GSC456 (**B**) cells expressing control shRNA, YB-1–specific shRNA, or YB-1 shRNA supplemented with CCT4 or mLST8. (**C** and **D**) Cell viability analysis of GSCWL1 (**C**) and GSC456 (**D**) cells described in **A** and **B**. Data are presented as mean ± SEM (*n =* 3). ***P < 0.001 by 1-way ANOVA followed by Dunnett’s test. (**E** and **F**) Relative numbers of tumor spheres formed in GSCWL1 (**E**) and GSC456 (**F**) cells described in **A** and **B** (*n =* 5). ***P < 0.001 by 1-way ANOVA with Dunnett’s test. (**G** and **H**) In vitro extreme limiting dilution assays were performed in GSCWL1 (**G**) and GSC456 (**H**) cells described in **A** and **B**.

A growing number of studies indicate that glioblastoma stem–like cells (GSCs) can recapitulate the heterogeneity and plasticity state of glioblastoma in vivo and are crucial for glioblastoma initiation, maintenance, and resistance to conventional therapies (47–49). We applied the GSC model to obtain a deeper understanding of the role of the YB-1/CCT4/mLST8/mTOR axis in glioma growth. We established GSC lines stably transduced with control shRNA, YB-1 shRNA, or YB-1 shRNA complemented with CCT4 or mLST8 expression plasmids. Compared with control shRNA, 2 independent YB-1 shRNAs markedly reduced CCT4 and mLST8 expression in GSCWL1 and GSC456 cells (Supplemental Figure 5, A and B), while exogenous expression of YB-1 increased CCT4 and mLST8 expression as well as endogenous YB-1 protein (Supplemental Figure 5, C and D), suggesting the existence of the YB-1/CCT4/mLST8/mTOR axis and autoregulation of YB-1 in GSCs. Knockdown of YB-1 substantially inhibited cell proliferation in GSCWL1 and GSC456 cells (Supplemental Figure 5, E and F) and reduced GSC frequency and self-renewal (Supplemental Figure 5, G–J). Reintroduction of CCT4 or mLST8 expression in YB-1–knockdown GSCWL1 and GSC456 cells reactivated mTOR signaling (Figure 6, A and B) and partially rescued cell proliferation, tumor-sphere formation, and GSC self-renewal (Figure 6, C–H). Collectively, these data indicate that the YB-1/CCT4/mLST8 axis is required for cell proliferation and the self-renewal of GSCs.

### The YB-1/CCT4/mLST8/mTOR axis promotes glioblastoma growth in vivo.

**Figure 7:**
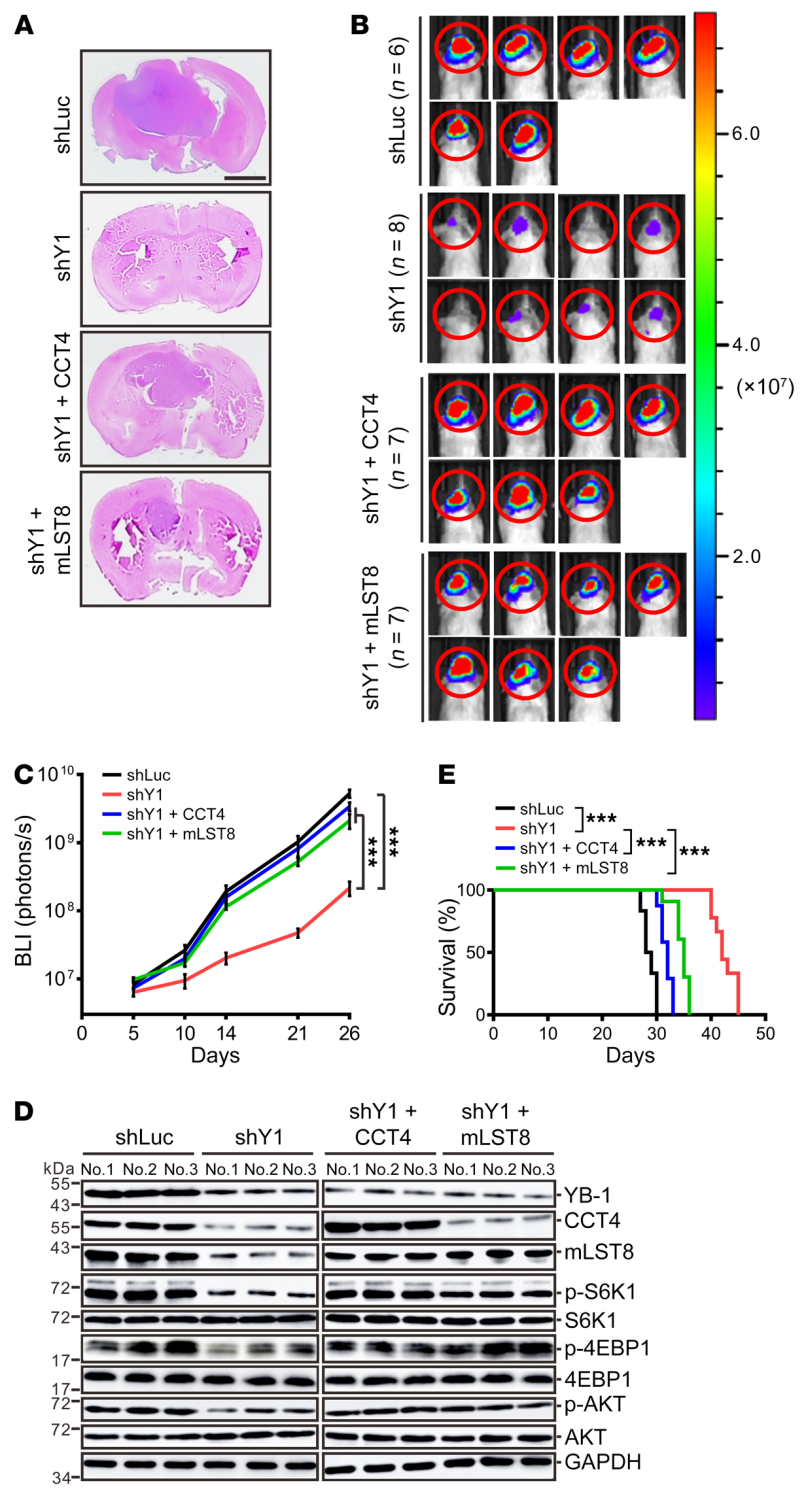
The YB-1/CCT4/mLST8 axis promotes tumor growth in vivo. (**A**) H&E-stained sections of tumor-bearing mouse brains intracranially injected with GSCWL1 cells expressing control shRNA, YB-1–specific shRNA, or YB-1 shRNA supplemented with CCT4 or mLST8. Scale bar: 2 mm. (**B**) Bioluminescence images of tumor-bearing mouse brains described in **A**. Colored scale bar represents photons/s/cm^2^/steradian. (**C**) Total flux (photons/s) was detected by bioluminescence imaging (BLI) at times indicated in mouse brains described in **A**. Data are presented as mean ± SEM. ***P < 0.001 by 2-way ANOVA. (**D**) Western blot analysis of YB-1, CCT4, mLST8, and mTOR markers in tumors derived from nude mice intracranially implanted GSCWL1 cells described in **A**. (**E**) Kaplan-Meier survival curves of nude mice intracranially implanted GSCWL1 cells described in **A**. ***P < 0.001 by Mantel-Cox log-rank test.

The above results indicated that YB-1 increases CCT4 translation, resulting in increased mLST8 folding/stability. Concordantly, overexpression of either CCT4 or mLST8 rescued the activity of mTORC1 and mTORC2 signaling in YB-1–knockdown cells (Figure 3, E and F, Figure 4, I and J, and Figure 6, A and B). To examine the functional significance of this pathway, we carried out nude mouse xenograft experiments. GSCWL1 and U87 cells infected by adeno-associated virus (AAV) carrying the luciferase coding sequence were used to establish stable cell lines expressing control shRNA, YB-1–specific shRNA, and YB-1–specific shRNA supplemented with CCT4 or mLST8 expression. YB-1–knockdown cells formed smaller tumors compared with control cells (Figure 7, A–C, and Supplemental Figure 6, A–C). Introduction of CCT4 or mLST8 into YB-1–knockdown cells partially rescued in vivo tumor cell growth (Figure 7, A–C, and Supplemental Figure 6, A–C) and the intratumoral activity of both mTOR1 and mTOR2 signaling (Figure 7D and Supplemental Figure 6D). Importantly, the mice injected with YB-1–knockdown cells had the longest survival, while increasing the expression of CCT4 or mLST8 in YB-1–depleted cells shortened the survival of mice that received YB-1–knockdown cells (Figure 7E and Supplemental Figure 6E). These results demonstrated that YB-1 enhances tumor growth via CCT4 and mLST8 in vivo.

### The YB-1/CCT4/mLST8/mTOR pathway is upregulated in patients with glioblastoma.

**Figure 8:**
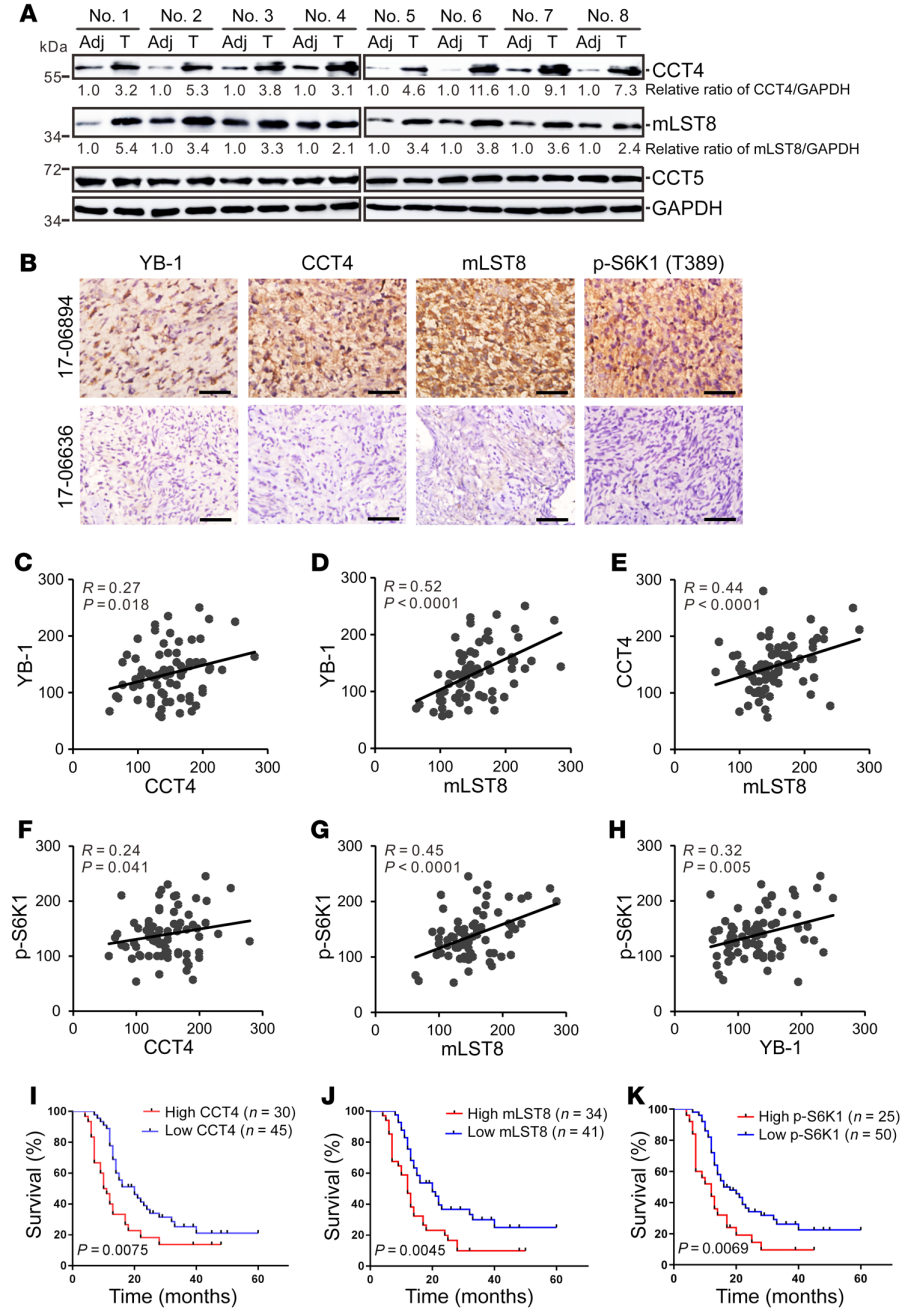
The YB-1/CCT4/mLST8/mTOR pathway is upregulated in glioblastoma. (**A**) Western blot analysis of CCT4, CCT5, mLST8, and GAPDH in 8 pairs of glioblastoma tumor tissues and their adjacent tissues. The normalized relative expression levels of CCT4 and mLST8 are shown below. (**B**) Representative images of IHC staining of YB-1, CCT4, mLST8, and phospho-S6K1 (T389) for 2 glioblastoma patients (patient 17-06894 with higher YB-1 expression, and patient 17-06636 with lower YB-1 expression). Scale bar: 10 μm. (**C**–**H**) Pearson’s correlation analysis between indicated proteins in 75 glioblastoma patients. *R* and *P* values were determined by Pearson’s correlation test. (**I**–**K**) Kaplan-Meier survival curves for glioblastoma patients with low (scores 0–150) versus high (scores 151–300) expression of CCT4, mLST8, and phospho-S6K1 proteins. *P* values were determined by Mantel-Cox log-rank test.

To investigate the biological significance of the YB-1/CCT4/mLST8/mTOR axis in glioblastoma, we first determined the expression of CCT4 and mLST8 in 8 pairs of glioblastoma tumor tissues (the same samples used in Figure 1A). Compared with adjacent tissues, both CCT4 and mLST8 were upregulated in glioblastoma tumor tissues, and CCT5, which was not affected by YB-1, did not show significant changes (Figure 8A). Next, we surveyed the expression of YB-1, CCT4, mLST8, and phospho-S6K1 (T389) using a cohort of glioblastoma patient samples (Supplemental Table 1) by performing IHC assays (Figure 8B). The expression levels of YB-1, CCT4, and mLST8 were mutually and positively associated with each other (Figure 8, C–E), and had a positive correlation with activated S6K1 signaling (Figure 8, F–H). Moreover, higher levels of CCT4, mLST8, and activated S6K1 predicted a poor survival, similarly to YB-1 (Figure 8, I–K), implying that YB-1 may serve as a promising target for the treatment of glioblastoma.

### Decoy oligonucleotides specifically binding to YB-1 inhibit glioblastoma growth in vivo.

**Figure 9:**
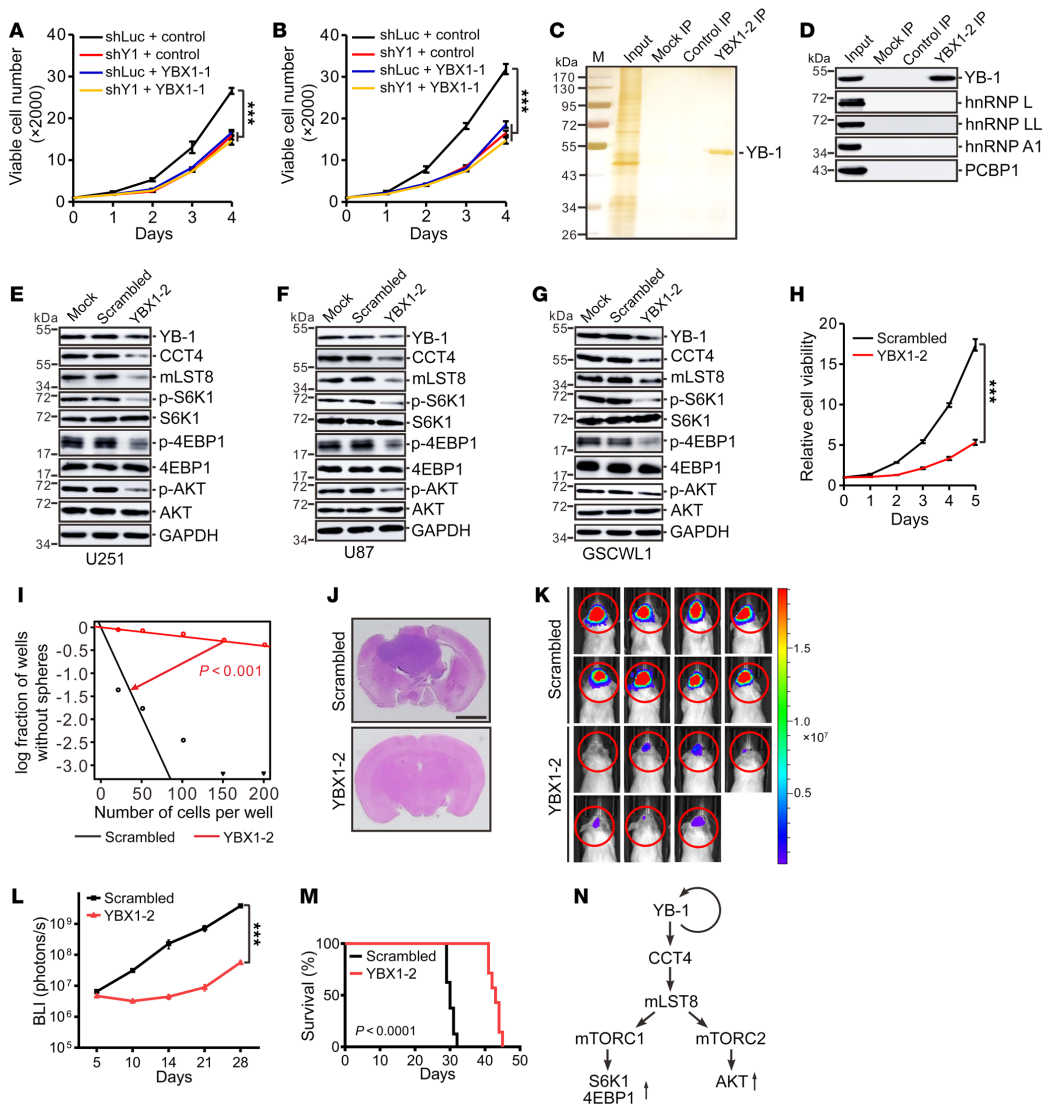
RNA decoy oligonucleotides targeting YB-1 inhibited tumor cell growth in vivo. (**A** and **B**) MTT analysis of cell growth in U251 (**A**) and U87 (**B**) cells expressing control or YB-1–specific shRNA with or without control or YB-1–specific (YBX1-1) decoy oligonucleotides. Data are presented as mean ± SEM (*n =* 3). ***P < 0.001 by 1-way ANOVA followed by Dunnett’s test. (**C**) SDS-PAGE of material pulled down without added oligonucleotides (mock) or with biotinylated scrambled or YB-1–specific (YBX1-2) decoy oligonucleotides followed by silver staining. (**D**) Western blotting for YB-1, hnRNP L, hnRNP LL, hnRNP A1, and PCBP1 in the material pulled down by biotinylated scrambled or YB-1 RNA decoy oligonucleotides from U87 cell extracts. (**E**–**G**) Western blot analysis of YB-1, CCT4, mLST8, and mTOR markers in U251 (**E**), U87 (**F**), and GSCWL1 (**G**) cells transfected with scrambled or YB-1 decoy oligonucleotides. (**H**) Effects of scrambled or YB-1 decoy oligonucleotides on cell proliferation were tested in GSCWL1 cells. Data are presented as mean ± SEM (*n =* 3). ***P < 0.001 by unpaired, 2-tailed Student’s *t* test. (**I**) In vitro extreme limiting dilution assays were performed in GSCWL1 cells transfected with scrambled or YB-1 decoy oligonucleotides. (**J**) H&E-stained sections of tumor-bearing mouse brains. Tumors were formed by intracranial injection of GSCWL cells transfected with scrambled or YB-1 decoy oligonucleotides (scrambled, *n =* 8; YBX1-2, *n =* 7). Scale bar: 2 mm. (**K**) Bioluminescence images of tumor-bearing mouse brains described in **J**. Colored scale bar represents photons/s/cm^2^/steradian. (**L**) Total flux (photons/s) was determined by bioluminescence imaging (BLI) for the times indicated after intracranial injection of GSCWL1 cells described in **J**. Data are presented as mean ± SEM. ***P < 0.001 by 2-way ANOVA. (**M**) Kaplan-Meier survival curves of nude mice described in **J**. *P* value was determined by Mantel-Cox log-rank test. (**N**) Schematic illustration of the working model.

Previously, we defined the RNA binding consensus of YB-1 as CAU/CC or UYAUC through in vitro SELEX and in vivo iCLIP-seq approaches (20, 29). The crystal structure of the CSD in complex with an RNA probe containing the CAUC sequence reveals that 4 highly conserved aromatic residues (W65, F74, F85, and H87) in YB-1’s CSD interact with CAUC mainly through π-π stacking interactions with high affinity (50). In an attempt to target YB-1, we designed RNA decoy probes that contain the CAUC sequence and used them to block the RNA binding activity of YB-1. As shown in Figure 9, A and B, transfection of RNA decoys carrying 1 copy of CAUC inhibited cell growth to a similar extent as YB-1 knockdown in U251 and U87 cells, and such inhibition required YB-1 protein since no further cell growth reduction was observed after YB-1 depletion, suggesting that these RNA decoys indeed repress the cell growth through targeting YB-1.

We further compared RNA decoys carrying 1 or 2 copies of the CAUC motif and found that RNA decoys with 2 copies of CAUC have a stronger effect than those with 1 copy (data not shown). Biotinylated RNA oligonucleotides carrying 2 copies of CAUC pulled down YB-1 specifically from cellular extracts of U87 cells, but not other RNA binding proteins that recognize C/A- or C-rich sequences (Figure 9, C and D). Notably, introduction of YB-1–specific RNA decoys carrying 2 copies of CAUC into cells inhibited both mTORC1 and mTORC2 signaling in U251, U87, and GSCWL1 cells (Figure 9, E–G). In addition, YB-1 RNA decoys inhibited the expression of YB-1, indicating that they are able to block the autoregulation of YB-1 (Figure 9, E–G). Notably, YB-1 RNA decoys inhibited cell proliferation and self-renewal of GSCWL1 cells (Figure 9, H and I). Importantly, the mice implanted with GSCWL1 or U87 cells transfected with YB-1 RNA decoys resulted in a slower tumor growth (Figure 9, J–L, and Supplemental Figure 7, A–C), an improved survival compared with those with scrambled oligonucleotides (Figure 9M and Supplemental Figure 7D), and a reduced intratumoral activity of both mTORC1 and mTORC2 (Supplemental Figure 7, E and F). Collectively, RNA decoy oligonucleotides recognizing YB-1 have an antiglioblastoma function through targeting YB-1 in vivo.

## Discussion

In this study, we discovered that YB-1 can function as a critical activator of mTOR signaling through mediating a self-activated pathway that impairs the protein homeostasis program in glioblastoma (Figure 9N). Our results showed that the level of YB-1 protein but not its mRNA is markedly elevated and predicts a poor prognosis. YB-1 activates mTOR signaling through promoting efficient folding of mLST8 via upregulation of CCT4 translation. The autoregulation of its own translation maintains YB-1 expression at a higher level and active mTOR signaling. This self-reinforced regulation pathway is abnormally activated in glioblastoma to support tumor progression, and thus targeting YB-1 with RNA decoys dramatically reduces tumor growth, providing evidence that YB-1 is potentially a good target for the treatment of glioblastoma.

PI3K/AKT/mTOR signaling is one of the most frequently activated pathways during the tumorigenesis of numerous malignancies, including glioblastoma, as a consequence of loss of PTEN or activating mutations found in the genes encoding PIK3CA and PIK3R1 (51). Thus, mTOR has been considered as a potential therapeutic target for glioblastoma treatment. However, the mTOR inhibitor rapamycin and its analogs have been ineffective in clinical trials, in part due to incomplete inhibition of mTORC1 and unexpected activation of mTOR via the loss of negative feedback loops (52). Understanding the regulation of mTOR signaling in glioblastoma may promote the development of novel strategies for targeting the mTOR pathway. Using RNA decoy oligonucleotide technology, we established the concept that targeting YB-1 inhibits the growth of glioblastoma in vivo.

In addition to the clinical relevance, this study provided mechanistic insights into cellular proteostasis, which is tightly controlled at different steps, including protein biogenesis, folding, assembly, localization, and degradation. We found multiple routes for modulating proteostasis directly or indirectly by an RNA binding protein, YB-1. First, YB-1 binds the 5′UTR of *CCT4* and its own mRNA, and increases the translation initiation of CCT4 and itself (Figure 5). Secondly, through CCT4, YB-1 enhances the folding and stability of mLST8, which is a substrate of the protein chaperone complex CCT (Figure 4). Thirdly, YB-1 promotes the translation of mTOR downstream targets indirectly by activating mTOR signaling. Previous studies showed that *YB-1* contains a terminal oligopyrimidine–like sequence and was downregulated by mTOR inhibitor Torin1, PP242, or INK128 in several cultured mammalian cell lines, suggesting that YB-1 might be a downstream target of the mTOR pathway (53–55). Although we did not observe a significant change in YB-1 expression after the treatment with mTOR inhibitors in glioblastoma cells (data not shown), these data suggest that besides YB-1 autoregulation, YB-1/CCT4/mLST8/mTOR might form a positive feedback loop leading to altered proteostasis networks in certain tissues.

The eukaryotic group II chaperonin CCT has been shown to play an important role in protein folding (56, 57). The CCT complex forms a double-ring structure. Each ring is composed of 8 paralogous subunits (CCT1–8) with a central cavity for positioning the substrate (58). CCT requires the binding and hydrolysis of ATP to induce conformational changes during the folding process (59). Cryoelectron microscopy analysis of the yeast CCT complex revealed that CCT4 is the last component in the complex to bind ATP during the step of CCT ring closure, suggesting that CCT4 serves as an ATP sensor and a rate-limiting component (60). Upregulation of CCT4 in glioblastoma may increase the recognition of certain substrates and accelerate conformational changes during the folding process. Initially, it was proposed that CCT recognizes its substrates through specific sequence determinants, such as the charged and polar residues found in the 2 well-characterized CCT substrates tubulin and actin (61). Using a combined proteomic and bioinformatic approach, approximately 300 CCT-interacting proteins, which are involved in a variety of cellular processes, were predicted as potential substrates of CCT (62). These substrate candidates tend to have β-strands and hydrophobic polypeptides with complex topologies and are enriched in components of oligomeric protein complexes. In mTOR complexes, 2 mTOR components, mLST8 and RAPTOR, contain β-propeller structures. A recent structural study reported that both mLST8 and RAPTOR are substrates for the CCT complex (63). However, in glioblastoma cells, we found that only the expression of mLST8 protein was regulated by CCT, but not RAPTOR (data not shown), suggesting that CCT promotes the folding of its substrate in a cell-type-dependent manner. Our findings highlight that the CCT complex plays a critical role in glioblastoma growth. It will be interesting to identify the full repertoire of CCT complex substrates, which will improve our understanding of the regulatory mechanisms at the protein level in glioblastoma.

RNA decoy oligonucleotides have the advantage of targeting existing cellular proteins directly and blocking the RNA binding activity of RNA binding proteins efficiently and quickly without interfering with their other activities. A previous study showed that RNA decoy oligonucleotides containing 3 or 4 tandem motif repeats can specifically inhibit the activity of several splicing factors in the nucleus (64). We used RNA decoys carrying 1 or 2 motif repeats (YBX1-1 or YBX1-2), which resulted in repression of the downstream targets or pathways of YB-1, suggesting that shorter oligonucleotides that are competent for delivery may also work efficiently. Future investigations that include optimizing the length, chemical structure, and dose of RNA decoys and using antisense oligonucleotides targeting YB-1 are warranted for therapeutic tests or combination therapy.

Intertumoral and intratumoral heterogeneity has been a major consideration for the treatment of glioblastoma. Individualized treatment based on tumor molecular classification holds promise to become a more effective therapeutic strategy than a universal approach. Glioblastoma can be subdivided into different subtypes based on genetic alterations, gene expression profiles, and epigenetic modifications (51, 65–68). Clinically related and gene-expression-based molecular subclasses of glioblastoma mainly include proneural, classical, and mesenchymal types (65, 68). We analyzed the genomic and proteomic data from a recent integrated study of 99 glioblastomas (69) and found that YB-1 protein was enriched in the classical subtype, suggesting that a subset of patients with the classical subtype might have better clinical benefit from targeting YB-1. Systematic characterization of the molecular features of glioblastoma with high YB-1 expression in large-scale cohort studies is necessary for the future development and implementation of an effective strategy for targeting YB-1. Taken together, our results show that we have identified the YB-1/CCT4/mLST8/mTOR axis as a contributor to glioblastoma growth and suggest a therapeutic approach to target this axis using competitive RNA oligonucleotides.

## Methods

### Cell culture.

U251, U87 (cell bank of the Chinese Academy of Sciences), and HEK293T cells (ATCC) were grown in Dulbecco’s modified Eagle’s medium (DMEM) supplemented with 10% fetal bovine serum. GSC456 cells were gifts from UCSD. The GSCs were cultured in stem cell medium consisting of DMEM/F12 supplemented with EGF and bFGF (20 ng/mL each), B27 without vitamin A, sodium pyruvate, and Glutamax. All cell culture reagents were purchased from Thermo Fisher Scientific. GSCs were isolated from surgical specimens or xenografts through fluorescence-activated cell sorting (FACS) and functionally characterized as previously described (70, 71). Briefly, tumors were dissociated with a Papain Dissociation System (Worthington Biochemical) according to the manufacturer’s instructions and recovered in the above-mentioned stem cell medium for at least 6 hours. GSCs were sorted using anti-CD133/1 antibody–conjugated magnetic beads (Miltenyi Biotec) followed by confirmatory assays for the expression of stem cell markers, including Sox2 and Olig2, sphere formation (in vitro limiting dilution assay), and secondary tumor initiation in immunocompromised mice. GSCWL1 cells were derived from a primary glioblastoma of a 55-year-old male patient.

### Cell transfection and reagent treatment.

U251 and U87 cells (both 2 × 10^5^) seeded in 35-mm culture dishes were transfected with a final concentration of 50 nM for siRNAs, 500 nM for YBX1-1, and 200 nM for YBX1-2 RNA decoys using Lipofectamine 3000 (Thermo Fisher Scientific). GSCWL1 and GSC456 cells (both 1 × 10^5^) seeded in 60-mm culture dishes were transfected with a final concentration of 200 nM RNA decoys using Lipofectamine RNAiMAX (Thermo Fisher Scientific). U251 cells were treated with CHX (100 μg/mL, Sigma-Aldrich) for 0, 1, 2, 4, 8, or 12 hours, MG132 (100 μM, Sigma-Aldrich) for 8 hours, or Baf A1 (200 nM, Sigma-Aldrich) for 24 hours.

### Oligonucleotides.

The sequences of all the oligonucleotides purchased from Invitrogen, Ribobio (siRNAs), or GenePharma (RNA decoys) for this study are listed in Supplemental Table 2.

### Plasmid construction.

To clone shRNA expression plasmids, primer pairs containing shRNA sequences were mixed, annealed, and inserted into pSIREN RetroQ or pLVX Lenti vectors between *Eco*RI and *Bam*HI. To clone FLAG-tagged CCT4 or mLST8 expression plasmids, PCR fragments encoding CCT4 or mLST8 were amplified from U251 cell cDNAs and inserted into the polylinker region of the pCDH-CMV-MCS-EF1-Puro vector between *Nhe*I and *Bam*HI. To clone GFP reporter plasmids, the 5′UTR and 3′UTR sequences of *CCT4* or *YB-1* were amplified from U251 cell cDNAs and inserted into the polylinker region of the pEGFP-N1 or pEGFP-C1 vector. Point mutations were introduced by a 2-step PCR method.

### Establishment of stable knockdown or overexpression cell lines using lentiviral or retroviral systems.

HEK293T cells were transfected with lentiviral or retroviral expression constructs together with respective helper plasmids using the calcium phosphate method. U251, U87, GSCWL1, and GSC456 cells were infected with recombinant viruses and selected for stable expression of FLAG-tagged proteins or shRNAs using puromycin according to the manufacturers’ instructions (System Biosciences and Clontech).

### MTT cell proliferation assay.

U251 and U87 cells were seeded at a density of 2000 per well in 24-well culture plates. After 24, 48, 72, 96, and 120 hours of incubation, cells were treated with 3-(4,5-dimethylthiazol-2-yl)-2,5-diphenyltetrazolium bromide (MTT, Sigma-Aldrich) at a final concentration of 0.5 μg/μL for 4 hours. The resulting formazan was solubilized with dimethylsulfoxide (DMSO), and the absorption was measured at 570 nm using a spectrophotometer (Thermo Fisher Scientific).

### Cell migration and invasion assay.

The cell migration assay was performed as previously described (72). For the cell invasion assay, the upper chamber was coated with Matrigel and then the assay was performed similarly to the migration assay.

### Cell viability and sphere-formation assay.

Cell viability was measured using a CellTiter-Glo kit (Promega) by plating GSCs at a density of 1000 cells per well in 96-well plates, with 3 replicate wells. Neurosphere-formation assays were performed by plating GSCs in 48-well plates at a density of 2000 cells per well, with 5 replicate wells. The number of tumor spheres with a diameter greater than 50 μm was counted 7 days after plating.

### In vitro extreme limiting dilution assay.

The GSCs were seeded on 96-well plates at 20, 50, 100, 150, and 200 cells per well, with 12 replicates each. Seven days after plating, the presence and number of neurospheres in each well were scored and counted. Extremely limiting dilution analysis was performed using software available at http://bioinf.wehi.edu.au/software/elda Three biological replicates from each GSC culture were plated.

### RT-qPCR.

Total RNA was extracted from cultured cells or patient tissues using TRIzol (Invitrogen) and reverse transcribed into first-strand cDNA using random hexamers by MMLV reverse transcriptase (Promega). PCR was performed using SYBR Green PCR Master Mix on a 7500 Fast Real-Time PCR system according to the manufacturer’s instructions (Applied Biosystems).

### Western blotting.

To extract the proteins from patient samples, adjacent and tumor tissues were homogenized in RIPA buffer containing 50 mM Tris-Cl pH 7.4, 150 mM NaCl, 1% NP-40, 0.1% SDS, 0.5% sodium deoxycholate, 1 mM EDTA-free protease inhibitor cocktail (Roche), and 1 mM phenylmethylsulfonyl fluoride (PMSF). Lysates were collected following the removal of insoluble material from tissue extracts by centrifugation at 20,817*g* for 20 minutes at 4°C, and then separated by SDS-PAGE followed by gel transfer to nitrocellulose membranes (Bio-Rad). The membranes were incubated first with primary antibodies, and then with secondary antibodies coupled to horseradish peroxidase (HRP). Band signals were detected with an enhanced chemiluminescence (ECL) system (Merck). Quantification of band intensity was performed using ImageJ software (NIH). The primary antibodies used for this study are anti-GAPDH (AC033, clone AMC0062), anti-CCT1 (A13364), anti-CCT3 (A6547), anti-CCT4 (A6548), anti-CCT5 (A6549), anti-CCT6 (A3589), anti-CCT7 (A12146), anti-CCT8 (A4449), anti-mLST8 (A1059), anti–pan S6K1 (A16658), anti-p62 (A0682), anti-PCBP1 (A1044), anti-FASN (A0461), anti-G6PD (A1537), anti-TPI1 (A2579), anti-PGAM1 (A4015), anti-DEPTOR (A9447), anti–pan 4EBP1 (A1248), anti–p-4EBP1 (T37/46; AP0030) from ABclonal; anti–hnRNP A1 (sc-32301, clone 4B10), anti–hnRNP LL (sc-132712), anti-CCT2 (sc-374152, clone D-8) from Santa Cruz Biotechnology; anti–YB-1 (Y0396), anti-FLAG (F3165, clone M2), anti-LC3B (L7543), anti–hnRNP L (R4903, clone 4D11) from Sigma-Aldrich; anti–p-S6K1 (T389; 9234S), anti–p-AKT (S473; 9271S), anti–pan AKT (4691P), anti-PKM2 (4053T), anti-RICTOR (2114T), anti-RAPTOR (2280T) from Cell Signaling Technology; anti–pan mTOR (66888-1-Ig, clone 1G11A3) from Proteintech; anti–p-mTOR [S2481; ab137133, clone EPR427(N)] from Abcam; and anti-GFP (11814460001, clones 7.1 and 13.1) from Roche. The HRP-conjugated secondary antibodies anti–mouse IgG (W4021) and anti–rabbit IgG (W4011) were purchased from Promega.

### Thermolysin treatment.

Cell lysates in buffer containing 50 mM Tris-Cl, pH 7.5, 50 mM KCl, 5 mM CaCl_2_, 5% glycerol, and 0.05% NP-40 were incubated with thermolysin (Sigma-Aldrich) at a final concentration of 150 μg/mL at 4°C. Reactions were stopped by the addition of 1 mM PMSF and 5 mM EDTA.

### RNA-seq and data analysis.

Total RNAs were processed for paired-end (2 × 150 nt) RNA-seq on an Illumina NovaSeq 6000 platform according to manufacturer’s instructions. Data analysis was carried out as previously described (73). Briefly, we used Trimmomatic (v0.39, http://www.usadellab.org/cms/?page=trimmomatic) to remove Illumina adapters and low-quality sequences. The trimmed reads were mapped to human reference genome hg38 using hisat2 (v2.2.1, http://daehwankimlab.github.io/hisat2) with default parameters. Then, the reads were counted for each gene by htseq-count, and differential expression was analyzed with the R package edgeR (v3.28.1, https://www.bioconductor.org/packages/3.10/bioc/html/edgeR.html). Genes with low counts were filtered by keeping only genes with rowSums (CPM[*y*] > 1) ≥ 2 and the logCPM from edgeR was converted to RPKM using the formula RPKM = 2^(logCPM – log2[gene length in kb])^. Custom R scripts were used to obtain significantly up- or downregulated genes that were defined by an FDR of less than 0.05, with FC greater than 2 and RPKM greater than 0.5 as cutoffs.

### Quantitative proteomics.

Cells were lysed with SDT lysis buffer (4% w/v SDS, 100 mM Tris-HCl, pH 7.6, 0.1 M DTT) and then heated for 5 minutes at 95°C, followed by sonication for 2 minutes (6 seconds on and 4 seconds off, power = 40 watts). After centrifugation for 5 minutes at 14,000*g*, the supernatant was collected in new tubes. Quantification of the protein extract was carried by a tryptophan-based fluorescence quantification method (74). The protein sample was then digested in 10 kDa centrifugal filter tubes (Millipore) via a filter-aided sample preparation protocol (75). Digestion was performed in 50 mM NH_4_HCO_3_ solution, and trypsin (Promega) was first added at a 1:50 trypsin-to-protein ratio and incubated for 12 hours at 37°C, followed by adding an equal trypsin amount for an additional 4 hours of incubation. After centrifugation at 12,000*g* for 10 minutes at room temperature, the peptide mixture was eluted into clean tubes and quantified using a Pierce BCA Protein Assay kit (Thermo Fisher Scientific). The peptide mixture was desalted by StageTips (3M Bioanalytical). Finally, the purified peptide samples were redissolved in 0.1% formic acid and quantified by NanoDrop 2000c spectrophotometer (Thermo Fisher Scientific). Equivalent peptides of each sample were analyzed on a Thermo Fisher Scientific Q Exactive HF hybrid quadrupole-Orbitrap mass spectrometer coupled with a Thermo Fisher Scientific EASY-nLC 1000 nanoflow LC. Samples were separated at a constant flow rate of 650 nL/min using a homemade microtip C18 column (75 μm × 250 mm) packed with ReproSil-Pur C18-AQ 2.4-μm resin (Dr. Maisch GmbH). Samples were resolved with the following gradients: 0 to 2 minutes, 2% to 4% buffer B (0.1% formic acid in acetonitrile); 2 to 104 minutes, 4% to 25% B; 104 to 114 minutes, 25% to 35% B; 114 to 116 minutes, 35% to 90% B; 116 to 120 minutes, 90% B. Xcalibur software was applied for data-dependent acquisition. A lock-mass *m*/*z* of 445.12003 was used for internal calibration. Electrospray voltage (2.8 kV) was applied and the capillary temperature was set at 320°C. MS scans were performed at 120 K resolution, collecting from 350 to 1500 *m*/*z* for 120 minutes (AGC target 3 × 10^6^, maximum ion time of 30 ms). The top 15 precursors were collected at 15 K resolution (AGC target 1 × 10^5^, maximum ion time of 35 ms) with an isolation window of 1.0 *m*/*z*, using 28% normalized collision energy. The exclude-isotope state was on, rejecting unassigned, 1+, 7+, 8+, and greater than 8+ ions with a dynamic exclusion time of 40 seconds. For quantification of cellular proteomic data, raw data searching was conducted in MaxQuant 1.6.0.16 (https://maxquant.org/) with default settings against the UniProt human database (https://ftp.uniprot.org/pub/databases/uniprot/previous_releases/release-2017_12/knowledgebase/; accessed December 20, 2017), combined with contaminant and decoy sequences. Carbamidomethylation of cysteine was set as fixed modification, in addition to oxidation of methionine and acetyl (protein N-terminus) as variable modifications. Each peptide was allowed no more than 5 modifications. Digestion mode set Trypsin/P as the specific enzyme and the maximal missed cleavage site was defined as 2. First-search peptide tolerance and main-search peptide tolerance were 20 ppm and 4.5 ppm, respectively. The searching criteria of proteins and peptides were at an FDR of less than 0.01. The minimal peptide length was set at 7 amino acids. R version 4.0.5 (http://www.R-project.org/) was used to carry out MS data normalization and statistical analysis. To identify significantly differential protein groups between control and YB-1–knockdown cells, we performed 2-sided Student’s *t* tests. *P* values less than 0.05 were considered significant.

### Immunoprecipitation.

Cells were lysed in 50 mM Tris-Cl, pH 7.5, 150 mM KCl, 0.5% NP-40, and 1 mM PMSF at 4°C for 30 minutes with rotation. Lysates were centrifuged at 17,949*g* for 10 minutes at 4°C. The supernatant was collected and incubated with anti–FLAG M2 beads (Sigma-Aldrich) or the appropriate antibodies that were previously immobilized on Protein G Dynabeads (Thermo Fisher Scientific) at 4°C for 3 hours with rotation. The beads were washed 3 times with the above-mentioned buffer.

### mLST8 interactome determined by immunoprecipitation coupled with MS analysis.

FLAG-mLST8 was immunoprecipitated with the anti–FLAG M2 beads from U251 cells stably expressing FLAG-mLST8. Beads were washed 3 times with 500 μL 100 mM NH_4_HCO_3_ followed by adding 20 μL 8 M urea in 100 mM Tris-Cl, pH 8.5 and sonication for 30 minutes. DTT at a final concentration of 10 mM (Sigma-Aldrich) and iodoacetamide at a final concentration of 10 mM (Sigma-Aldrich) for reduction and alkylation were added to the solution and incubated at 56°C and room temperature, respectively, for 30 minutes. For optimizing the activity of trypsin, the protein mixture was diluted 4-fold and digested with trypsin at 1:50 (w/w) (Promega). The digestion was stopped by adding formic acid to 5% final concentration. The peptide mixture was desalted by use of a monospin C18 column (SHIMADZU-GL), and dried out by speed vacuum. All the raw files from Q-Exactive were searched against the UniProt human database (released on October 22, 2015 and accessed on October 15, 2020) using the Integrated Proteomics Pipeline v3.1 (https://www.manula.com/manuals/ip2/ip2/1/en/topic/3-1-logging-on). Precursor and product ion spectra were searched with an initial mass tolerance of 50 ppm and 600 ppm, respectively. Tryptic cleavage was selected, and up to 3 missed cleavages were allowed. Carbamidomethylation of cysteine (+57.02 Da) was set as a fixed modification, and oxidation (+15.99 Da) of methionine was set as a variable modification. The target-decoy-based strategy was applied to control both peptide and protein-level FDRs lower than 0.01.

### RNA immunoprecipitation.

Cells were irradiated with UV light at 150 mJ/cm^2^ and lysed in buffer containing 50 mM Tris-HCl, pH 7.4, 100 mM NaCl, 1% NP-40, 0.1% SDS, 0.5% sodium deoxycholate, and protease inhibitor cocktail. RNAs were partially fragmented using RNase A (QIAGEN). After centrifuging at 10,000*g* for 10 minutes, an aliquot (10%) of supernatant was removed and served as input. The remaining supernatant was immunoprecipitated with either rabbit IgG or anti–YB-1 antibody immobilized on Protein G Dynabeads. The bound RNAs were washed extensively and isolated using TRIzol (Invitrogen) followed by RT-qPCR.

### Biotinylated-RNA pull-down assay.

Streptavidin beads (Thermo Fisher Scientific) were washed using binding/washing buffer (10 mM Tris-Cl, pH 7.5, 1 M NaCl, 1 mM EDTA) and then incubated with biotinylated RNA oligonucleotides for 15 minutes at room temperature. Cells were lysed in buffer containing 50 mM Tris-Cl, pH 7.5, 150 mM KCl, 0.2% NP-40, and 1 mM PMSF. Cell lysates were centrifuged at 17,949*g* for 10 minutes at 4°C. Then the supernatant was collected and incubated with biotinylated RNA immobilized on streptavidin beads for 2 hours at room temperature. The bound proteins were analyzed by Western blot analysis or silver staining. Silver staining was performed according to the manufacturer’s instructions (Beyotime Biotech).

### Polysome profiling assay.

Polysome profiling was carried out as previously described (76, 77). Briefly, control and YB-1–knockdown U251 cells were pretreated with 200 μM CHX for 5 minutes at 37°C, and lysed in polysome lysis buffer (100 mM KCl, 5 mM MgCl_2_, 10 mM HEPES, pH 7.4, 100 μg/mL CHX, 1× protease inhibitor cocktail, 100 U/mL RNase inhibitor, 25 U/mL Turbo DNase I, 2 mM DTT, 0.5% Triton X-100, and 0.5% sodium deoxycholate). Debris was removed by centrifugation at 15,294*g* for 10 minutes at 4°C, and supernatants were loaded onto 10-mL continuous 10% to 50% sucrose gradients (100 mM KCl, 5 mM MgCl_2_, 10 mM HEPES, pH 7.4, 100 μg/mL CHX, 1× protease inhibitor cocktail, and 100 U/mL RNase inhibitor) and centrifuged at 35,000 rpm for 2.5 hours at 4°C in an SW41 rotor (Beckman). Fractions were collected using a density gradient fractionation system (Brandel). Total RNA from each fraction was isolated using TRIzol (Invitrogen) and used for further analysis.

### IHC analysis of glioblastoma specimens and survival analysis.

The tissue sections from paraffin-embedded glioblastoma specimens were stained with antibodies against YB-1, CCT4, mLST8, and p-S6K1 (Millipore; T389; MABS82). The protein expression in tissue sections was evaluated using a standard scoring system (H-score) according to the staining intensity and the percentage of positive cells. The staining intensity was scored as 0, 1, 2, or 3, corresponding to negative, weak, moderate, or strong staining, respectively. The score was calculated by the formula 3 × (% strong staining) + 2 × (% moderate staining) + 1 × (% weak staining), giving a range of 0–300. Microscopic evaluation was carried out by 3 observers who were blinded to clinical and laboratory data. The scores were compared with overall survival, defined as the time from the date of diagnosis to death or last known date of follow-up.

### Intracranial xenograft assay in nude mice.

U87 and GSCWL1 cells (both 5 × 10^5^) were intracranially injected into the left cerebral cortex of 8-week-old male NOD-SCID mice at the following coordinates: M/L, –2.0 mm; A/P, 0 mm; and D/V, 2.75 mm. Bioluminescence imaging was conducted to monitor tumor growth using the IVIS Spectrum CT imaging system (PerkinElmer). Tumor-bearing mice were injected with D-luciferin (PerkinElmer) before anesthesia. Radiance (photons/s/cm^2^/steradian) was measured using Living Image 4.5.4 software (PerkinElmer).

### Data availability.

The RNA-seq data are available in NCBI’s Gene Expression Omnibus database (GEO GSE161523). The quantitative MS proteomics and protein interactome data have been deposited in the ProteomeXchange Consortium (http://proteomecentral.proteomexchange.org) via the iProX partner repository with the data set identifier PXD022776.

### Statistics.

For most in vitro assays, experiments were performed at least in triplicate. Data are presented as mean ± SEM, and unpaired, 2-tailed Student’s *t* tests or 1-way ANOVA followed by Dunnett’s test were used to calculate *P* values. Two-way ANOVA tests were used to evaluate in vivo tumor growth of different test groups. Survival curves were plotted using the Kaplan-Meier method and *P* values were determined by Mantel-Cox log-rank test. *P* less than 0.05 was considered statistically significant. Statistical analyses were performed using GraphPad Prism software.

### Study approval.

All brain tumor tissues were collected from informed, consenting patients in Huashan Hospital affiliated to Fudan University (Shanghai, China) from July 2013 to August 2018 with the approval from the Institutional Research Ethics Committee. All mice were treated according to the protocols approved by the Institutional Animal Care and Use Committee of the CAS Center for Excellence in Molecular Cell Science, Chinese Academy of Sciences.

## Author contributions

JH conceived the project. JH, LY, ZW, JZW, and HZ designed experiments. JZW, HZ, PY, HL, YY, YZ, and QL performed experiments. WKW and XF performed bioinformatics analysis. KX, PW, CP, RZ, and CCLW performed proteomic analysis. JH, ZW, LY, JZW, HZ, YH, and KL interpreted and analyzed data. YS, NZ, and XW provided advice on experiments. JH, JZW, HZ, and ZW wrote the manuscript with comments from all authors.

## Supplementary Material

Supplemental data

## Version 1 03/03/2022

In-Press Preview

## Version 2 04/15/2022

Electronic publication
