# Supplementary material for: Upregulated YB-1 protein promotes glioblastoma growth through a YB-1/CCT4/mLST8/mTOR pathway
Source: J Clin Invest. 2022 Apr 15;132(8):e146536. doi: 10.1172/JCI146536 (PMC9012288; doi:10.1172/JCI146536)
Supplement: Supplemental data [file jci-132-146536-s094.pdf]

Up-regulated YB-1 protein promotes glioblastoma growth through an YB-1/CCT4/mLST8/mTOR pathway

Jin-Zhu Wang,[1] Hong Zhu,[1] Pu You,[2] Hui Liu,[1] Wei-Kang Wang,[1] Xiaojuan Fan,[3] Yun Yang,[3] Keren Xu[4], Yingfeng Zhu,[5] Qunyi Li,[6] Ping Wu,[7] Chao Peng,[7] Catherine C. L. Wong,[7,13] Kaicheng Li,[2] Yufeng Shi[8], Nu Zhang[9], Xiuxing Wang[10], Rong Zeng[4], Ying Huang,[11] Liusong Yang,[12] Zefeng Wang,[3] and Jingyi Hui[1]

Supplemental data

7 Supplemental Figures

2 Supplemental Tables

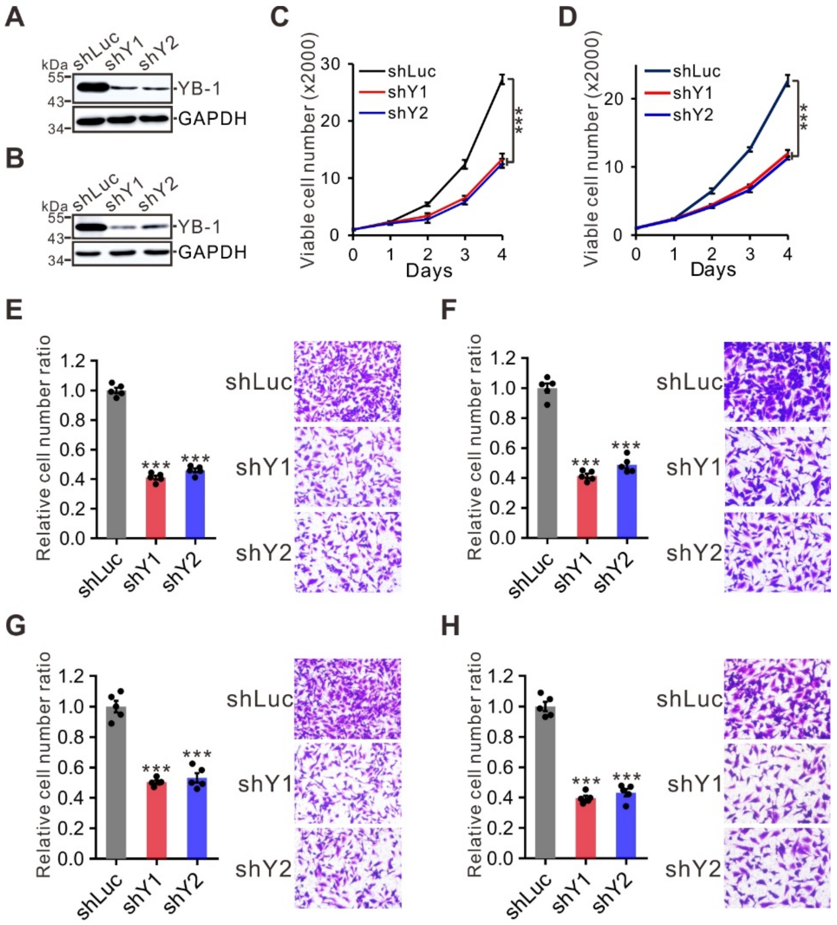

Supplemental Figure 1. Knockdown of YB-1 in glioblastoma cell lines inhibits cell proliferation, migration and invasion. (**A** and **B**) Western blot analysis of YB-1 expression in U251 (**A**) and U87 (**B**) cells expressing control- (shLuc) or two YB-1-specific (shY1 and shY2) shRNAs. (**C** and **D**) MTT analysis of cell growth in control- or YB-1-knockdown U251 (**C**) and U87 (**D**) cells (n=4). (**E** and **F**) Transwell analysis of cell migration in control- or YB-1-knockdown U251 (**E**) and U87 (**F**) cells (n=5). (**G** and **H**) Transwell analysis of cell invasion in control- or YB-1-knockdown U251 (**G**) and U87 (**H**) cells (n=5). Data are presented as mean ±SEM. *** P<0.001, by 1-way ANOVA followed by Dunnett's test (**C**-**H**).

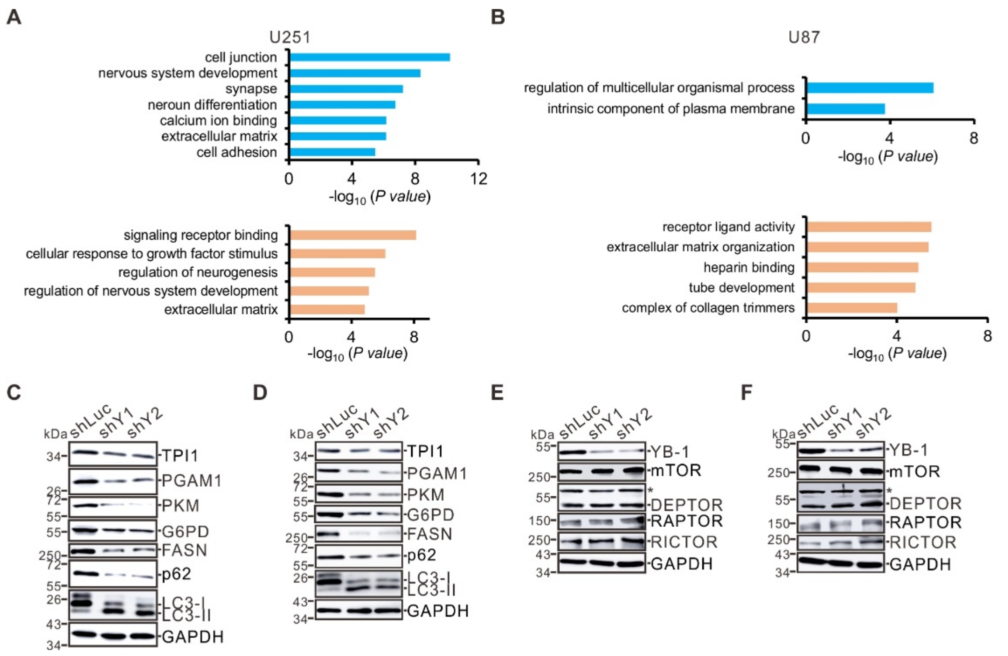

Supplemental Figure 2. Transcriptomic and proteomic analyses of gene expression changes upon YB-1 deletion in glioblastoma cells. (**A** and **B**) GO enrichment analysis of down-regulated (blue) or up-regulated (orange) mRNAs induced by YB-1 knockdown in U251 (**A**) and U87 (**B**) cells. (**C** and **D**) Validation of quantitative mass spectrometry analysis of control- or YB-1-knockdown U251 (**C**) and U87 (**D**) cells by Western blotting. (**E** and **F**) Western blot analysis of mTOR, DEPTOR, RAPTOR, and RICTOR in control- or YB-1-knockdown U251 (**E**) and U87 (**F**) cells. * indicates a non-specific band.

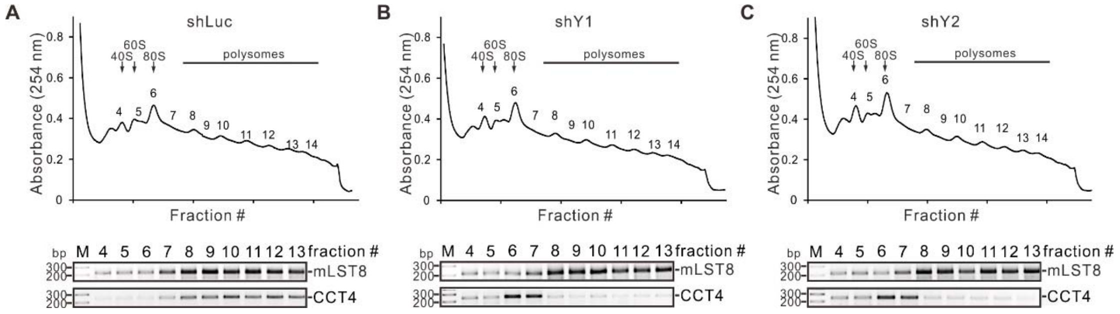

Supplemental Figure 3. Detection of *mLST8* and *CCT4* mRNAs in polysome profile fractions. RT-PCR analysis of the association of *mLST8* and *CCT4* mRNAs with ribosomes and polysomes fractionated on sucrose gradients using U251 cells expressing control- (**A**), shY1 (**B**) or shY2 (**C**) shRNAs.

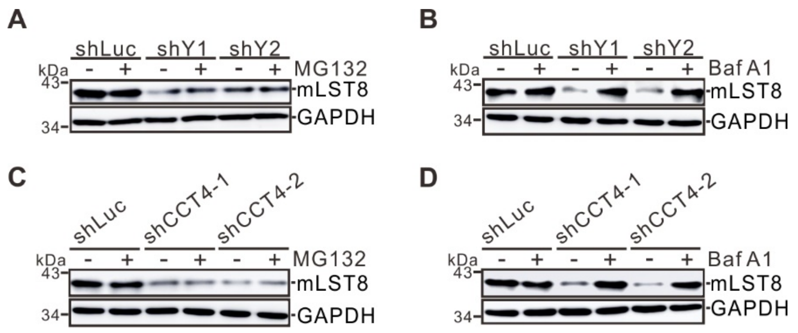

Supplemental Figure 4. Knockdown of YB-1 or CCT4 induces mLST8 degradation by lysosome. (**A** and **B**) Western blot analysis of mLST8 in control- or YB-1-knockdown U251 cells treated with or without MG132 (**A**) or Baf A1 (**B**). (**C** and **D**) Western blot analysis of mLST8 in control- or CCT4-knockdown U251 cells treated with or without MG132 (**C**) or Baf A1 (**D**).

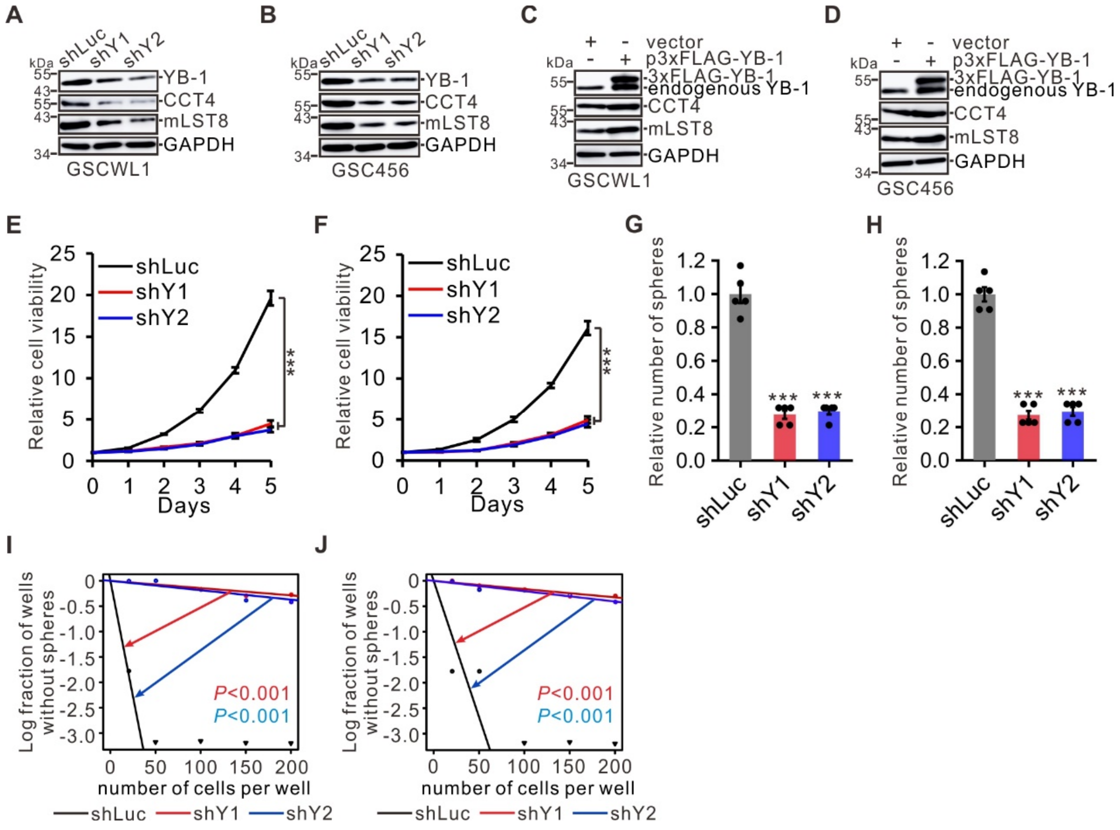

Supplemental Figure 5. YB-1 is required for cell proliferation and self-renewal of GSCs. (**A** and **B**) Western blot analysis of YB-1, CCT4, and mLST8 in GSCWL1 (**A**) and GSC456 (**B**) cells expressing control or YB-1-specific shRNAs. (**C** and **D**) Western blot analysis of YB-1, CCT4, and mLST8 in GSCWL1 (**C**) and GSC456 (**D**) cells exogenously expressing FLAG-tagged YB-1. (**E** and **F**) Cell viability of GSCWL1 (**E**) and GSC456 (**F**) cells described in **A** and **B** (n=3). *** P<0.001, by 1-way ANOVA followed by Dunnett's test. (**G** and **H**) Relative numbers of tumorspheres formed in GSCWL1 (**G**) and GSC456 (**H**) cells described in **A** and **B** (n=5). *** *P*<0.001, by 1-way ANOVA followed by Dunnett's test. (**I** and **J**) In vitro extreme limited dilution assays were performed in GSCWL1 (**I**) and GSC456 (**J**) cells described in **A** and **B**.

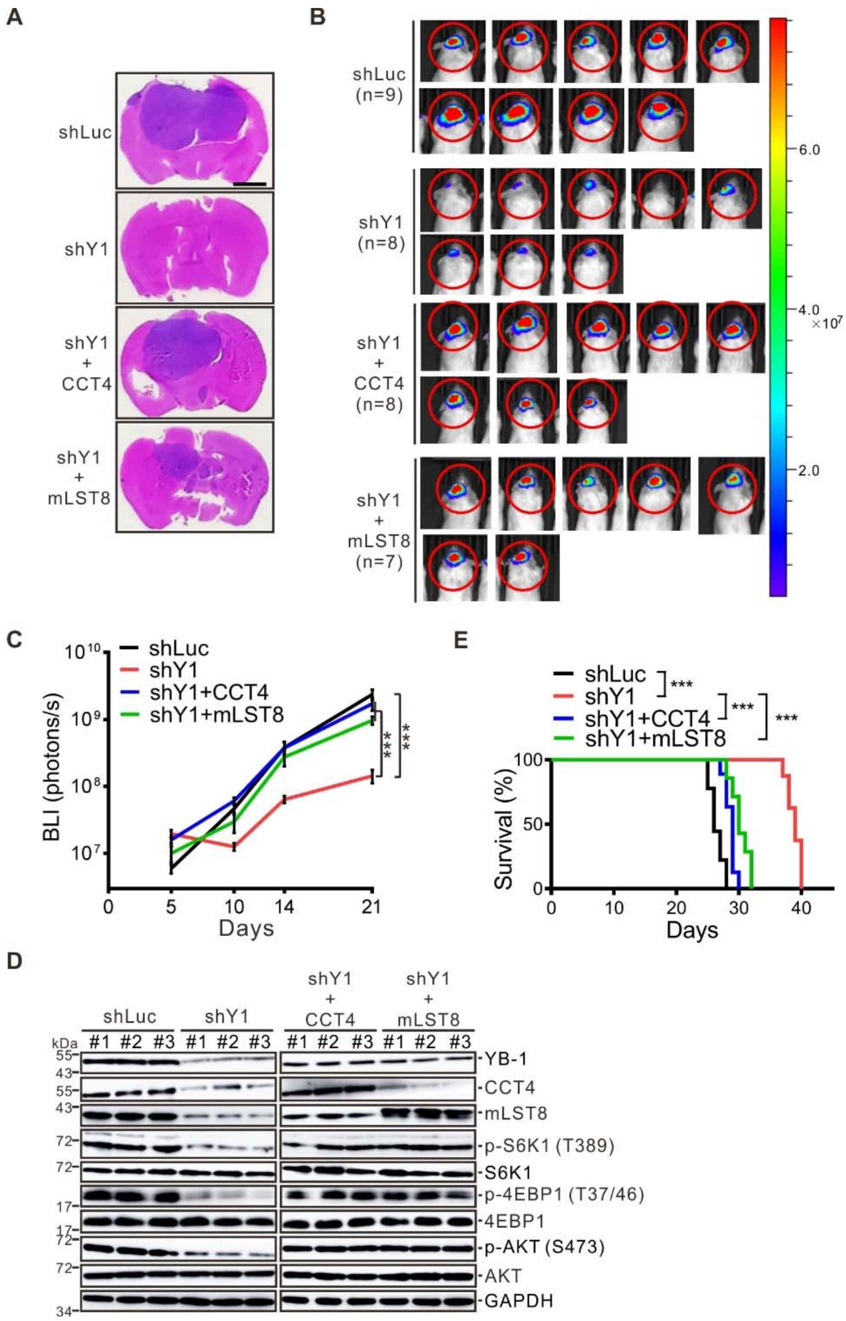

Supplemental Figure 6. The YB-1/CCT4/mLST8 axis promotes tumor formation of U87 cells in vivo. (**A**) H&E-stained sections of tumor-bearing mouse brains intracranially injected with U87 cells expressing control shRNA, YB-1-specific shRNA, or YB-1 shRNA supplemented with CCT4 or mLST8. Scale bar, 2 mm. (**B**) In vivo bioluminescent imaging of tumor-bearing mouse brains described in **A**. Colored scale bar represents photons/s/cm^2/steradian. (**C**) Total flux (photons/s) was detected at time indicated after U87 cells described in **A** were injected intracranially into nude mice. Data are presented as mean ±SEM. *** $P<0.001$, by 2-way ANOVA test. (**D**) Western blot analysis of YB-1, CCT4, mLST8, and mTOR markers in tumors derived from nude mouse brains intracranially implanted with U87 cells described in **A**. (**E**) Kaplan-Meier survival curves of nude mice intracranially transplanted U87 cells described in **A.** *** $P<0.001$, by Cox-Mantel log-rank test.

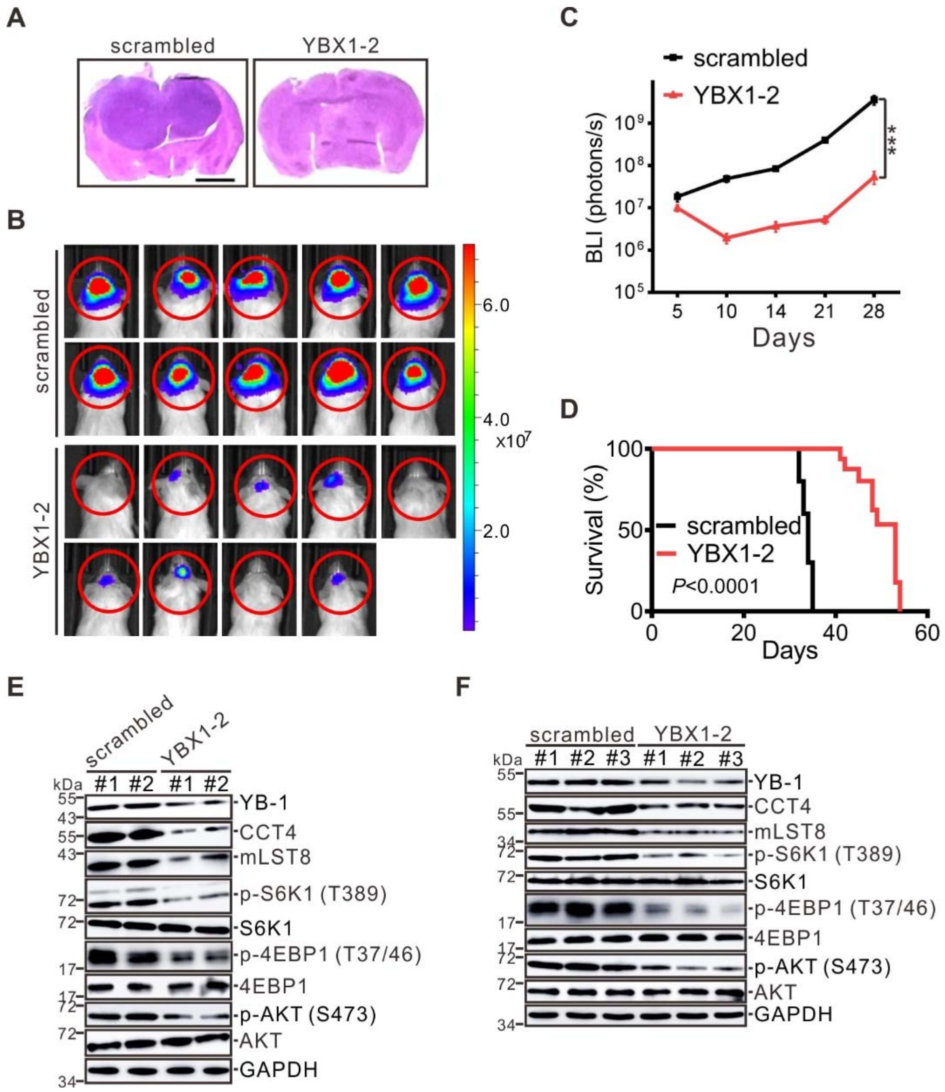

Supplemental Figure 7. RNA decoy oligonucleotides targeting YB-1 inhibited tumor cell growth in vivo. (**A**) H&E-stained coronal section of tumor-bearing mouse brains. Tumors were formed by intracranial injection of U87 cells transfected with the scrambled or YB-1 decoy oligonucleotides. Scale bar, 2 mm. (**B**) In vivo bioluminescent imaging of tumor-bearing mouse brains described in **A** (scrambled: n=10; YBX1-2: n=9). Colored scale bar represents photons/s/cm^2/steradian. (**C**) Total flux (photons/s) was determined for the time indicated after intracranial injection of U87 cells described in **A**. Data are presented as mean ±SEM. *** P<0.001, by 2-way ANOVA test. (**D**) Kaplan-Meier survival curves of nude mice described in **A**. P value was determined by Cox-Mantel log-rank test. (**E** and **F**) Western blot analysis of YB-1, CCT4, mLST8, and mTOR markers in tumors derived from nude mouse brains implanted with U87 (**E**) or GSCWL1 (**F**) cells transfected with the scrambled or YB-1 decoy oligonucleotides.

Supplemental Table 1. The clinical and molecular data of 75 glioblastoma patients[a] in this study

Features	High YB-1 n=23	Low YB-1 n=52	Test of significance
Histology			
Primary	23	52	
Recurrent	0	0	
Sex			
Male	12	36	χ^2=2.014
Female	11	16	*P*=0.1559
Age			
<50	6	20	χ^2=1.078
≧50	17	32	*P*= 0.2992
IDH1 status			
WT	18	35	χ^2=3.468
Mutant (R132H)	2	16	*P*=0.06257
IDH2 status			
WT	20	51	
Mutant (R172K)	0	0	
MGMT methylation			
Unmethylated	3	6	
+ (25%)	6	10	
++ (50%)	5	14	χ^2=2.216 *P*=0.6961
+++ (75%)	4	9	
++++ (100%)	2	12	

Note:

a. Four GBM patients in this cohort are missing molecular data.

Supplemental Table 2. Sequences of oligonucleotides used in this study

Oligonucleotide name	Oligonucleotide sequences (5'-3')
shLuc-F	GATCCCGTACGCGGAATACTTCGATTCAAGAGATCGAAGTATTCCGCGTACGTTTTTT GCTAGCG
shLuc -R	AATTCGCTAGCAAAAAACGTACGCGGAATACTTCGATCTCTTGAATCGAAGTATTCC GCGTACGG
shY1-F	GATCCGGTCATCGCAACGAAGGTTTTCAAGAGAAACCTTCGTTGCGATGACCTTTTT TGCTAGCG
shY1-R	AATTCGCTAGCAAAAAAGGTCATCGCAACGAAGGTTTCTCTTGAAAACCTTCGTTGC GATGACCG
shY2-F	GATCCAGAAGGTCATCGCAACGAATTCAAGAGATTCGTTGCGATGACCTTCTTTTTT TGCTAGCG
shY2-R	AATTCGCTAGCAAAAAAAGAAGGTCATCGCAACGAATCTCTTGAATTCGTTGCGATG ACCTTCTG
shCCT4-2-F	GATCCCCCTATGTGTTATTCGTTGTTTTCAAGAGAAACAACGAATAACACATAGGG TTTTTTGCTAGCG
shCCT4-2-R	AATTCGCTAGCAAAAAACCCTATGTGTTATTCGTTGTTTCTCTTGAAAACAACGAATA ACACATAGGGG
shCCT4-3-F	GATCCGCTTTCTCCAATGAGTGTAAATTCAAGAGATTTACACTCATTGGAGAAAGCT TTTTTGCTAGCG
shCCT4-3-R	AATTCGCTAGCAAAAAAGCTTTCTCCAATGAGTGTAAATCTCTTGAATTTACACTCAT TGGAGAAAGCG
shmLST8-1-F	GATCCGGAAACTGCTATGTCTGGAATTCAAGAGATTCCAGACATAGCAGTTTCCTTTT TTGCTAGCG
shmLST8-1-R	AATTCGCTAGCAAAAAAGGAAACTGCTATGTCTGGAATCTCTTGAATTCCAGACATA GCAGTTTCCG
shmLST8-2-F	GATCCGCCAGCGGATCTTCCAGGTGAACTTCAAGAGAGTTCACCTGGAAGATCCGC TGGCTTTTTTGCTAGCG
shmLST8-2-R	AATTCGCTAGCAAAAAAGCCAGCGGATCTTCCAGGTGAATCTCTTGAAGTTCACCTG GAAGATCCGCTGGCG
CCT4-5'UTR-BamH1-F	TTAAAGGATCCGGCAAGGAAGGCCCCCTTCTCCGCCT
CCT4-5'UTR-Not1-R	AGAGTCGCGGCCGCTTTACTTGTACAGCTCGTCCAT
CCT4-5'UTR-overlap-F	GTGAGCAAGGGCGAGGAGCTGTTCACCGGGGT
CCT4-5'UTR-overlap-R	CAGCTCCTCGCCCTTGCTCACTGCCACATTCTCGGGCATGGCAAACTC
CCT4-5'UTR-Mut-F	ACCGTTATTGCGCTGCGGCCGGCCAGAATCCGGGT
CCT4-5'UTR-Mut-R	ACCCGGATTCTGGCCGGCCGCAGCGCAATAACGGT
CCT4-3'UTR-BsrG1-F	TTAAATGTACAAGTAATCTGGATAACTGACTAGCACCATTATGATCACCAGT
CCT4-3'UTR-BamH1-R	TTAAAGGATCCTATGTATACACTTTTATTTGCAGAAGGAATCC
YB-1-5'UTR-BamH1-F	TTAAAGGATCCATTCTCGCTAGTTCGATCGG TAGCGGGAGC
YB-1-5'UTR-Not1-R	AGAGTCGCGGCCGCTTTACTTGTACAGCTCGTCCAT
YB-1-5'UTR-overlap-F	ATGGTGAGCAAGGGCGAGGAGCTGTT
YB-1-5'UTR-overlap-R	CAGCTCCTCGCCCTTGCTCACCATGGTTGCGGTGATGGTGACTGGGGCCGGCT

YB-1-5'UTR-Mut-F	CGCCGCCGCCTCCGGCCTAGTTACCGTCGCTCCCCGGGAGGAGCCGCAGCT
YB-1-5'UTR-Mut-R	CCGGGGAGCGACGGTAACTAGGCCGGAGGCGGCGGCGGGGCTGCTCAGGGCTCTCT
YB-1-3'UTR-BsrG1-F	TTAAATGTACAAGTAAATGCCGGCTTACCATCTCTACCATCAT
YB-1-3'UTR-BamH1-R	TTAAAGGATCC ATTATTTAAGACCTTTATTAACAGGT
pCDH-CCT4-Nhe1-FLAG-F	TTAAAGCTAGCATGGACTACAAGGACGACGATGACAAGCCCGAGAATGTGGCACCC
pCDH-CCT4-BamH1-R	TTAAAGGATCCTTATCGAGTGTTTACCACATCATCTATTTTCAGAAT
pCDH-XbaI-mLST8-F	TTAAATCTAGAATGGACTACAAGGACGACGATGACAAG
pCDH-Not1- mLST8-R	TTAAAGCGGCCGCCTAGCCCAGCACACTGTCATTGAAGGCCAG
mLST8-qPCR-F	ACGCACCCATTAACTGCGTG
mLST8-qPCR-R	CGTCAGATTCCAGACATAGCAG
CCT4-qPCR-F	ATTCAGTTTTGCTTATCTGCTCC
CCT4-qPCR-R	TTCAATATCCTTAATCACCATGATC
YB-1-qPCR-F	ATGCCGGCTTACCATCTCTACC
YB-1-qPCR-R	TCAACGGGCAAAAAGCAAGC
actin-qPCR-F	TGGACTTCGAGCAAGAGATG
actin-qPCR-R	GTGATCTCCTTCTGCTGCATCCTG
siRNA- CCT4 -1	CCCUAUGUGUUAUUCGUUGUU(dTdT)
siRNA- CCT4 -2	GCUUUCUCCAAUGAGUGUAAA(dTdT)
siRNA-mLST8-1	GGAAACUGCUAUGUCUGGAA(dTdT)
siRNA-mLST8-2	GCCAGCGGAUCUUCCAGGUGAAC(dTdT)
control-oligo	GUCGUCUU (each base with 2'-O-methoxyethyl modifications)
scrambled-oligo	AGAUCUCGUUCGUAUUCUUA (each base with 2'-O-methoxyethyl modifications)
YBX1-1-oligo	GUCAUCUU (each base with 2'-O-methoxyethyl modifications)
YBX1-2-oligo	GUCAUCUUGAUAGUCAUCUU (each base with 2'-O-methoxyethyl modifications)
